# Interrelations of work with health and wellbeing on a 50+ year old workforce assessed using longitudinal self-reports and actigraphy

**DOI:** 10.1038/s41598-026-58229-z

**Published:** 2026-07-28

**Authors:** Athanasios Tsanas, Belinda Steffan, Billy Dixon, Kiersten Hay, Larissa Pschetz, Jakov Jandric, Wendy Loretto

**Affiliations:** 1 Usher Institute, University of Edinburgh, Usher Building, 5-7 Little France, Edinburgh, EH164UX UK; 2 School of Mathematics, University of Edinburgh, Edinburgh, UK; 3 Business School, University of Edinburgh, Edinburgh, UK; 4 Design Informatics, University of Edinburgh, Edinburgh, UK

**Keywords:** Aging, Workers in mid-later life, Self-reported outcome measures, Wearable sensors, Wellbeing, Actigraphy, Health care, Psychology, Psychology, Risk factors

## Abstract

Daily activities, work, and health are closely intertwined. We closely followed 45 participants aged 50–66 years working across different sectors in a year-long study, who completed a range of baseline, weekly, and monthly Participant Reported Outcome Measures (PROMs). Participants self-reported on work-related aspects using the Brief Job Stress Questionnaire (BJSQ) and Workplace Wellbeing Question Bank (WWQB); wellbeing using the Warwick-Edinburgh Mental Wellbeing Scale (WEMWBS); and sleep using the Pittsburgh Sleep Quality Index (PSQI). Additionally, they provided wrist-worn actigraphy data collected over three periods (each period ~ 35–40 days). In total, we collected 1901 PROMs entries and 5258 days of actigraphy data. We computed 55 actigraphy measures broadly assessing physical activity, sleep, and diurnal variability characteristics. We explored statistical associations between pairs of PROMs items and between PROMs items with actigraphy measures using correlations and cross-correlations to assess similarities. We built mixed-effects models to associate PROMs items and actigraphy measures with global PSQI and total WEMWBS. We found very strong associations (>|0.5|) between BJSQ items with total WEMWBS and global PSQI, highlighting certain work aspects affect sleep and overall wellbeing, whereas actigraphy analysis provided new insights uncovering sleep quality problems including disturbances and awakenings for most participants. Additionally, the mixed-effects models revealed that actigraphy-derived sleep measures are strongly associated with total WEMWBS and global PSQI providing complementary information to PROMs. Integrating longitudinal work-related self-reports and actigraphy can provide useful personalized wellbeing- and health-insights, and should likely be considered in future smart-health monitoring systems to promote healthy aging.

**Supplementary Information:**

The online version contains supplementary material available at 10.1038/s41598-026-58229-z.

## Introduction

The population is growing older globally and current estimates project a quarter of the EU population will be 65 years old and over by 2050^1^. Similar statistics are reported for the UK population, where about 17.7 million people will be aged over 65 years by 2050 (https://www.ons.gov.uk/peoplepopulationandcommunity/birthsdeathsandmarriages/ageing). In relation to the workforce, one in three workers in the UK are over 50, a figure which reached a record high in 2023 of 3.6 million, expected to rise further (https://www.gov.uk/government/statistics/economic-labour-market-status-of-individuals-aged-50-and-over-trends-over-time-september-2023/economic-labour-market-status-of-individuals-aged-50-and-over-trends-over-time-september-2023). Increasing age is strongly associated with increased morbidity and use of healthcare resources, with population aging and an associated aging workforce posing a major challenge to health and care systems worldwide^1,2^. The underlying rationale of our study is exploring how day-to-day living activities, work, and health are intertwined in older workers’ lives over the long-term, with a view to identifying potentially modifiable risk factors.

Low levels of Physical Activity (PA) and prolonged sedentary behaviors such as sitting, combined with social isolation, increase the risk of many chronic conditions, premature onset of ill health and frailty^3,4^. A key challenge is supporting older people to live healthy, independent lives for as long as possible, and maintaining PA and fitness is central to this^5^. The workplace is a key environment influencing the PA and health of the over-50 workforce, particularly given their often extensive work engagement and long working hours. Modifying some working patterns or habits to increase PA levels might be an expedient and cost-effective intervention to positively influence health.

Similarly, a further challenge is to enable and support active participation in paid work for people over 50^1^: health problems are one of the main reasons that older workers opt for early voluntary retirement^6,7^. However, with the right workplace supports in place, older workers are more likely to remain economically active for longer^6^. Given that health problems may lead to early retirement and that workplace factors can influence health, protecting the health of older workers by improving the workplace is a key strategy to extend their working lives and address the aging workforce. This has a range of important implications in terms of the workplaces’ broader potential societal and economic impact beyond individuals’ physical and mental health.

Indicatively, a recent systematic review synthesized evidence on understanding the causes and effects of work on workers’ health and wellbeing, revealing factors such as work-related stress, the nature of the work, and workplace interactions potentially having a major detrimental effect^8^. Furthermore, a recent review study reported how a range of characteristics of individual workplaces, such as interpersonal and teamwork factors along with employee behaviors, are associated with health and wellbeing^9^. They emphasized in particular the dynamic aspects of work and health noting that fluctuations may occur within days or weeks, which tacitly indicates that the interrelations between work and health should be monitored *longitudinally*. Additionally, prior studies highlight the need to understand person-specific trajectories: similarly to the precision medicine paradigm focusing on individuals to evaluate healthcare outcomes, the same principle applies to understanding work-related aspects tailored to each individual^10^.

Traditionally, longitudinal assessments of wellbeing have relied on Participant Reported Outcome Measures (PROMs) which nowadays are increasingly elicited using smartphone apps^11,12^. The use of digital technology to obtain PROMs over paper-based questionnaire responding facilitates obtaining responses which are time-stamped^13^, and encourages regular engagement e.g. via prompts that may come in the form of a text-message or sound on the smartphone. Nevertheless, PROMs are by their nature subjective, require *active* participant input which might be challenging to retain longitudinally thus resulting in poor adherence and, if not done regularly, may be prone to recall bias^14,15^. Health-related PROMs have been widely deployed applications to understand symptoms and longitudinal trajectories in clinical outcomes^11,12^: work-focused and daily-living-focused PROMs can be similarly elicited to understand individuals’ *perception* of work-related and daily living effects.

Digital technologies have considerable potential towards providing *continuous* insights into both physical and mental wellbeing over and above PROMs, via *passively* collected information from different types of sensors^11,16–18^. Although the COVID-19 pandemic had considerable negative impact on levels of PA as well as mood, anxiety and sleep^19,20^, it has generally increased older people’s use of digital technology^21^. Given that some of these technologies, such as certain smartwatches, are becoming increasingly more affordable and are used by older people, some studies call for healthcare professionals to embrace digital healthcare technologies overcoming societal agism^21^.

Wrist-worn wearables in particular are reportedly unobtrusive and convenient to use including by older adults^22,23^. Some recent research-grade products enable data collection for over three months on a single charge (e.g. Axivity AX6, https://axivity.com/files/resources/AX6_Datasheet.pdf), which makes them ideal for longitudinal healthcare monitoring. The data modalities that can be collected from smartwatches vary, although typically research-grade wrist-worn sensors typically record three-dimensional acceleration, ambient light, and wrist temperature: from these modalities we can infer PA, sleep, and diurnal rhythm patterns^24–26^. Wrist-worn wearables have been used in large scale studies such as the UK BioBank (100,000 participants, 40–69 year-old), where the collected actigraphy data has provided novel insights into PA patterns and their association with chronic healthcare conditions^26,27^, and even linking sleep patterns with mortality^28^. Likewise, the “All of Us” research program in the US has very recently released data from 59,000 participants, and their platform enables bona fide researchers linkage to further resources such as Electronic Health Records (EHRs)^29^. These large-scale wearable studies are very useful from a population level/epidemiology perspective linking wearables and health, however they often have some limitations regarding device standardization (e.g. in “All of Us”), lack of longitudinal raw actigraphy data (UK BioBank), or having a targeted carefully screened population group. Smaller scale specialized studies understandably have more controlled settings, and have consistently reported how actigraphy-derived PA, sleep, and diurnal rhythm patterns provide clinically useful insights into specific conditions and rehabilitation monitoring over and above PROMs^24,30–32^. All in all, digital technologies (in particular smartwatches) have been largely embraced and could be deployed to complement insights gained from PROMs and/or other linked information e.g. EHRs, towards a more holistic understanding of daily living patterns and health-related outcomes.

Collectively, building on current evidence it is crucial to understand person-specific patterns of physical and mental health wellbeing in older workers, and there is strong evidence linking those to the workplace. While traditional self-reports, such as diaries and PROMs, provide essential insights into a person’s lived experience, they are often limited by subjectivity and inconsistent adherence. Integrating sensor-based data addresses these challenges by providing continuous, objective information. Consequently, combining PROMs with wrist-worn actigraphy offers a powerful strategy for exploring the complex ‘work-health relationship’. The aim of this study is to provide new insights into the interrelations between work-related aspects and wellbeing of the 50+ year-old workforce over the course of a year capitalizing on: (i) a range of regularly collected self-reports on standardized questionnaires and (ii) information that is extracted from wrist-worn actigraphy data regarding PA, sleep, and diurnal rhythm patterns.

## Results

### Assessing adherence

**Fig. 1:**
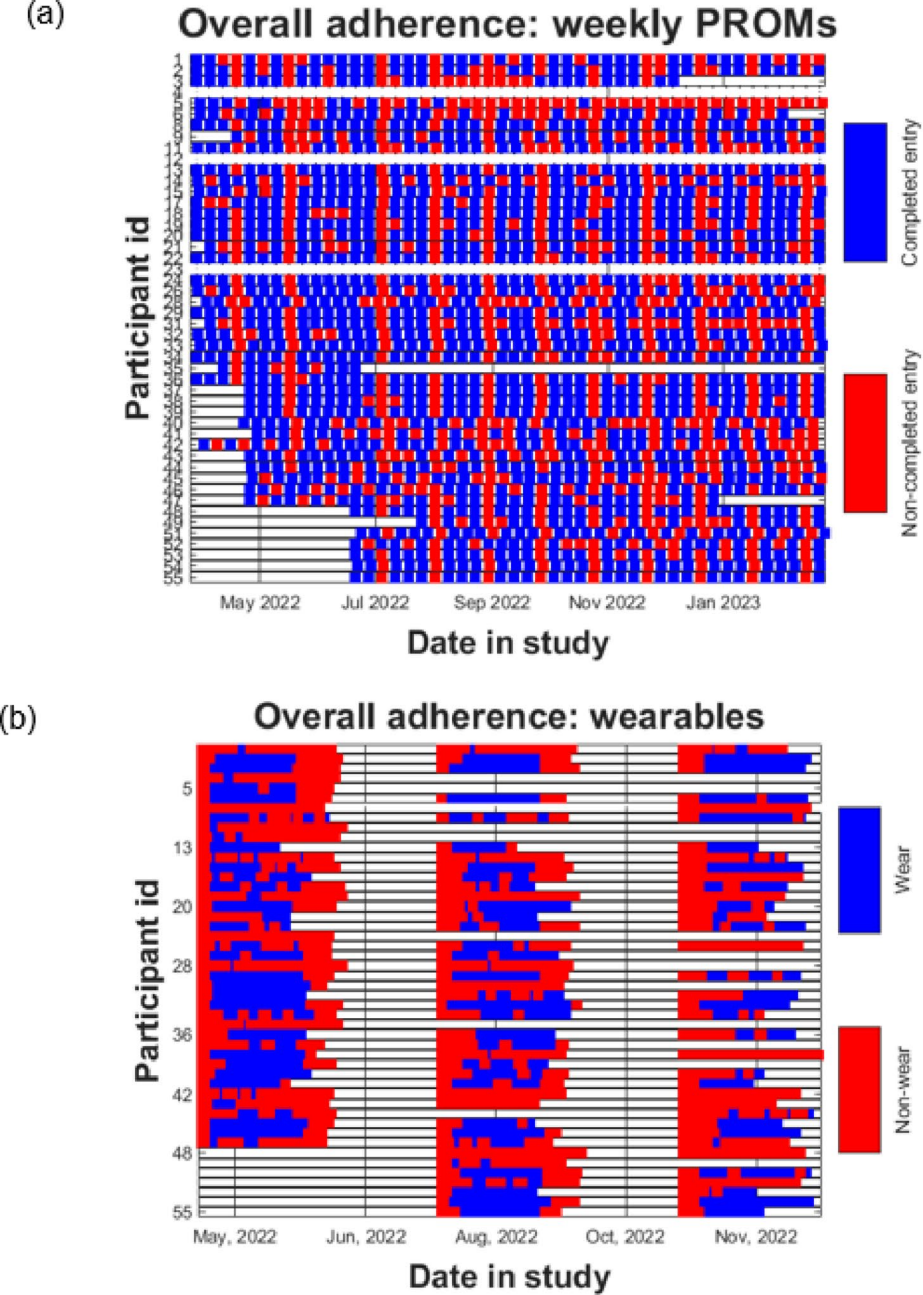
Longitudinal adherence for (**a**) weekly PROMs, (**b**) the wrist-worn wearable (Geneactiv). Blue indicates that an entry was successfully completed within 2 days from the expected completion date; otherwise that entry was marked as missing, denoted with red. For the wearable data we considered that a participant was adherent and provided “valid” data when they wore the device for at least 20 h in that day (marked with blue).

In total, we collected 1901 PROMs entries and 5258 days of actigraphy data across all participants. Figure 1 presents the overall adherence for the weekly PROMs and the wrist-worn wearable sensor. The PROMs adherence was 67 ± 12% (median ± interquartile range), and is striking to note that there were some weeks that participants almost consistently across the cohort did not register PROMs, e.g. in mid-May, mid-July and early September. See also Supplementary Fig. S1 for the running adherence on weekly PROMs completion displaying the percentage of completed entries for each participant throughout the study. Following visual inspection of Fig. 1a and Supplementary Fig. S1 most participants exhibited a decline in PROMs adherence roughly after 3 months of participation, although almost everyone remained engaged until the end of the study with very few terminating participation early.

The adherence for the wearable sensors varied across participants (median participant adherence = 67%, if we exclude participants 8 and 42 who did not wear the device at all). We remark that the missing entries for the first few days for each participant are due to the device being sent through normal mail service (the devices start recording from the time they are removed from the configuration dongle at the University premises, so until they reach participants we typically do not have valid data for 2–3 days). Therefore, the actual adherence (for the engaged participants) from the day that they received the device is probably closer to 70%.

### Baseline PROMs and work sector differences

**Fig. 2:**
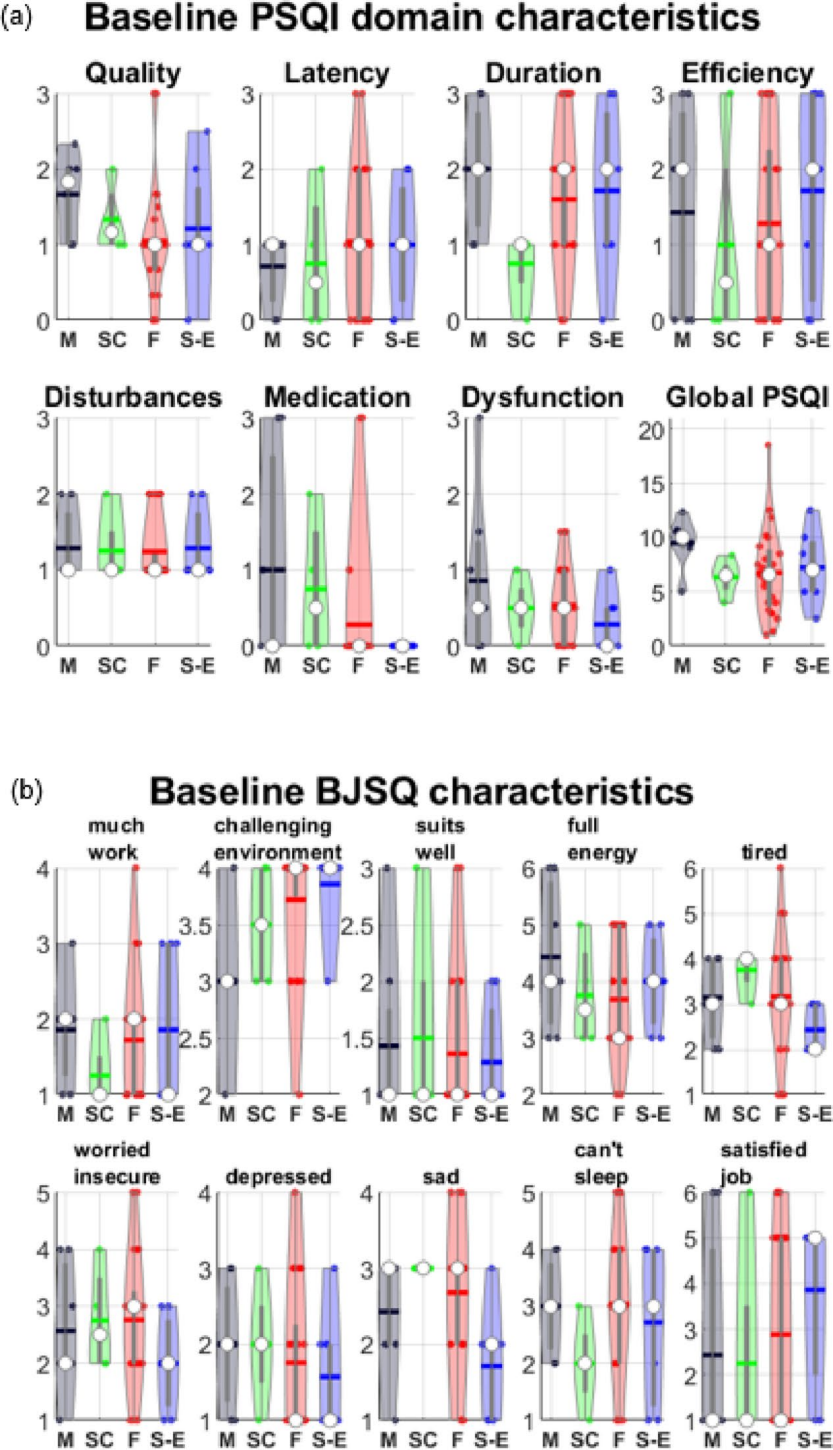

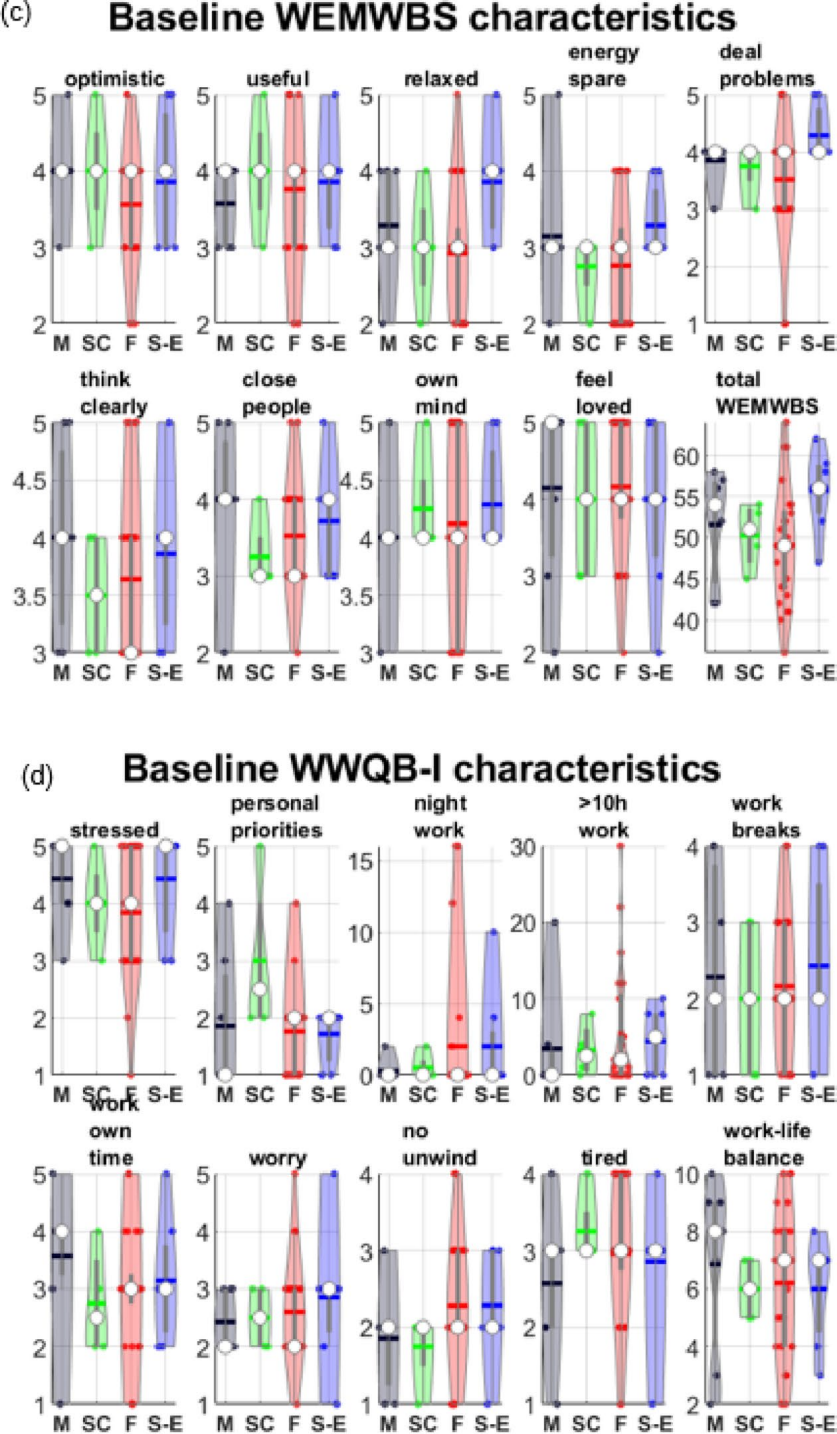

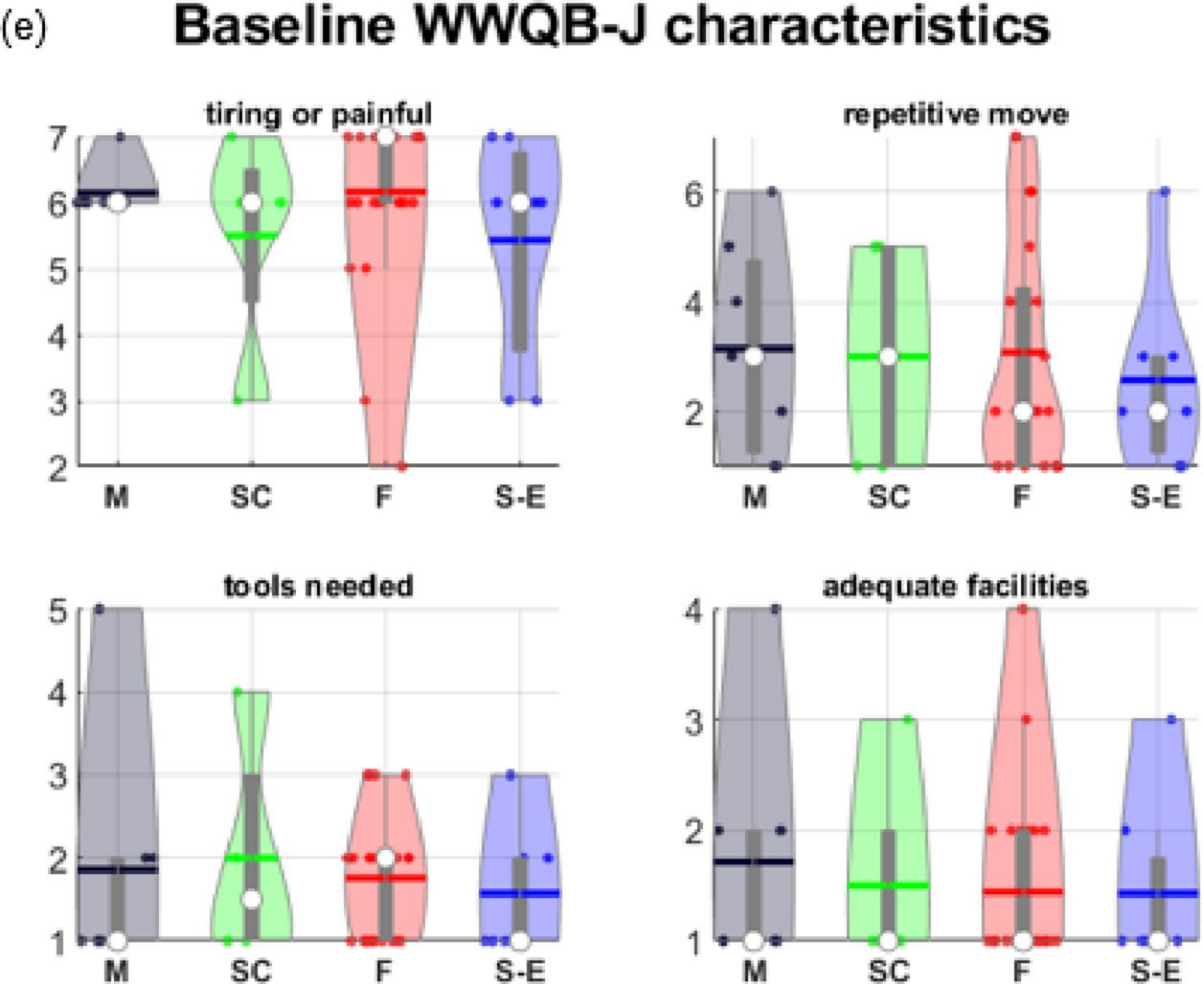
Violin plots presenting the baseline characteristics across the different questionnaires: (**a**) PSQI, (**b**) BJSQ, (**c**) WEMWBS, (**d**) WWQB-I, and (**e**) WWQB-J. For PSQI, we present the seven domains and the global PSQI score. For questionnaires comprising more than 10 items, we selectively present here 10 indicative items (and total score, where applicable) for clarity of illustration. We grouped participants across the four sectors they worked in: Manufacturing (M), Social Care (SC), Finance (F), Self-Employed (S-E). The white dot in each violin plot denotes the median, the horizontal line denotes the mean, the transparent vertical line spans from the 25th to the 75th percentile, and the dots are the individual samples. Readers may want to refer to the Supplementary Material titled “Questionnaires_in_SHAW.xlsx” file for item details.

Subsequently, we present the baseline PROMs across the four sectors in the study, which serve to understand the characteristics of the participant cohort when they started participating in the study (see Fig. 2). We remark that the global Pittsburgh Sleep Quality Index (PSQI) was high for all groups, indicating poor sleep for the vast majority of participants in the study (PSQI was above the usual threshold of 5 denoting sleep problems) regardless of the sector they work in. In general, participants reported considerable problems in terms of sleep quality, duration, and efficiency.

From the Brief Job Stress Questionnaire (BJSQ) we can infer there was variability in terms of workload across all four sectors with participants reporting they had a large amount of work, although the work environment was not considered challenging. Participants indicated they were largely satisfied with their jobs, with the exception of the self-employed group. Consistent with the PSQI self-reports, participants noted regular problems with sleep in BJSQ.

Very few participants scored highly in the Warwick-Edinburgh Mental Wellbeing Scale (WEMWBS), with total WEMWBS generally lower than 60. Participants working in finance had much greater WEMWBS variation compared to participants working in other sectors, with the median total WEMWBS comparatively lower. Most people scored highly in the items “feeling loved” and “dealing with problems”, however they reported moderate levels of energy to spare or feeling relaxed, which resonates with the level of tiredness in BJSQ.

In terms of the work-related aspects self-reported in the Workplace Wellbeing Question Bank (WWQB) item pool (WWQB-I assessing working time quality, and WWQB-J, assessing the physical environment at work), most participants indicated they have the tools and facilities needed at their work, and have time to attend to their personal priorities. The majority of participants indicated feeling moderately tired, which was consistent across all four sectors. Work-life balance was much more diverse, both within each sector and across sectors, and was not scored particularly high. Participants working in finance had the greatest variation, closely reflecting the total wellbeing as assessed using WEMWBS.

There are several ways to stratify and visualize the data to explore potential differences of interest; in Supplementary Fig. S2 we explored whether there are gender differences across the questionnaires.

### Exploring associations within and between questionnaires

The next step was to explore the inter- and intra-associations of different instruments (i.e. relationships between the questionnaires), in particular to associate work-related aspects and wellbeing. Supplementary Fig. S4 presents the heatmaps for all questionnaires used at baseline, weekly and monthly to provide an overview of these statistical relationships. We clarify that for PSQI we used the domains rather than the raw PSQI items, as per standard practice; and also in addition to the individual items, we have included the global PSQI and total WEMWBS.

Table 1 summarizes the strongest associations within (pairs of items of) the same questionnaire and also between (pairs of items of) the different questionnaires for the baseline PROMs. We remark that in some cases a single item may be highly associated with the total score (e.g. for PSQI and WEMWBS). As could be reasonably expected intuitively (and verified by inspecting Supplementary Fig. S4), the magnitude of correlations within questionnaires are typically higher than the correlations between different questionnaires. The bounds were tight around the computed correlation coefficients, which inspires confidence these are stable strong statistical relationships given that the number of samples used for these computations is relatively low (the number of participants). Overall sleep (expressed via the global PSQI) had been most strongly correlated with sleep duration. When participants reported they felt good, that was reflected in being confident and overall wellbeing (expressed via the total WEMWBS). Crucially, the total WEMWBS was very strongly associated with how people felt at work, for example if participants felt tired, gloomy, or irritable because of their jobs.

**Table 1:** Summary of notably strong associations for pairs of items within the same questionnaire (top part of Table) and between pair of items for the different questionnaires used (bottom part of Table) for the baseline PROMs.

	Item in questionnaire (1)	Item in questionnaire (2)	Correlation coefficient
Items within the same questionnaire	PSQI 1: sleep quality	Global PSQI	0.77 ± 0.03 [0.72, 0.82]
	PSQI 3: sleep duration	Global PSQI	0.81 ± 0.02 [0.79, 0.85]
	BJSQ 19: full energy	BJSQ 20: lively	0.87 ± 0.01 [0.87, 0.90]
	BJSQ 21: angry	BJSQ 22: annoyed	0.80 ± 0.03 [0.76, 0.88]
	BJSQ 21: angry	BJSQ 23: irritable	0.82 ± 0.02 [0.79, 0.86]
	BJSQ 32: not concentrating	BJSQ 33: gloomy	0.70 ± 0.03 [0.66, 0.75]
	BJSQ 16: job suits me well	BJSQ 56: satisfied with job	0.78 ± 0.02 [0.72, 0.80]
	WEMWBS 8: feel good	WEMWBS 10: feel confident	0.74 ± 0.02 [0.70, 0.78]
	WEMWBS 8: feel good	Total WEMWBS	0.76 ± 0.03 [0.72, 0.81]
	WEMWBS 10: feel confident	Total WEMWBS	0.73 ± 0.04 [0.68, 0.79]
	WEMWBS 14: cheerful	Total WEMWBS	0.75 ± 0.02 [0.74, 0.81]
Items between different questionnaires	BJSQ 27: tense	WEMWBS 3: relaxed	− 0.73 ± 0.03 [− 0.78, − 0.67]
	BJSQ 16: job suits me well	WWQB-I 10: work-life balance	− 0.40 ± 0.05 [− 0.46, − 0.30]
	PSQI 7: daytime dysfunction	Total WEMWBS	− 0.33 ± 0.05 [− 0.47, − 0.31]
	BJSQ 16: job suits me well	Total WEMWBS	0.38 ± 0.05 [0.28, 0.45]
	BJSQ 20: lively	Total WEMWBS	0.56 ± 0.03 [0.54, 0.67]
	BJSQ 23: irritable	Total WEMWBS	− 0.61 ± 0.05 [− 0.68, − 0.52]
	BJSQ 33: gloomy	Total WEMWBS	− 0.68 ± 0.03 [− 0.73, − 0.64]
	BJSQ 56: satisfied with job	Total WEMWBS	− 0.47 ± 0.05 [− 0.53, − 0.38]
	BJSQ 57: satisfied with family	Total WEMWBS	− 0.54 ± 0.04 [− 0.49, − 0.60]
	WWQB-I 9: tired	Total WEMWBS	− 0.52 ± 0.04 [− 0.56, − 0.43]
	WWQB-I 10: work-life balance	Total WEMWBS	0.49 ± 0.04 [0.39, 0.53]

We kept the description of each item using a short version above for brevity; see the corresponding questionnaires on how each item was exactly phrased when participants completed the entries ("Questionnaires_in_SHAW.xlsx"). We used the term ‘item’ freely above to refer both to the actual PROMs items provided by participants and also the overall computed scores (e.g. global PSQI). The last column is the Spearman correlation coefficient along with the standard deviation and confidence intervals. All reported correlations were statistically significant (*p* < 0.05), with bold entries having statistical significance *p*-values < 0.001. The computations used a single entry per participant from the baseline PROMs completion.

### Longitudinal PROMs trajectories

Figure S3 presents the trajectories of the weekly and monthly PROMs for an indicative participant, as an illustration of the underlying nature of the time series data (longitudinal PROMs) in the study. We use this as an exemplar to build a participant portrait on the basis of the provided PROMs (see the Supplementary Material for further information).

Next we wanted to evaluate joint associations between PROMs collected longitudinally. In Supplementary Fig. S5 we present global PSQI and total WEMWBS to visually inspect these relationships for five indicative participants across the study (chosen to illustrate the point of the variability in the underlying relationship between global PSQI and total WEMWBS for selected different participants). There is no consistent pattern across participants overall, a finding that becomes clearer in Supplementary Fig. S6 summarizing the cross-correlation coefficients (XCFs) in a scatter plot for all study participants to quantitatively express the variability of these statistical associations. A key finding from the computed XCFs was that there was considerable variability across participants in the observed relationships between pairwise item questionnaire comparisons. For example, we found that the XCFs between global PSQI and total WEMWBS varied from strongly proportional to strongly inversely proportional (Supplementary Fig. S6). This further motivates the need to be developing personalized approaches, and also complementing PROMs with additional data (e.g. actigraphy) to understand participant trajectories.

### Longitudinal insights from actigraphy complementing PROMs

**Fig. 3:**
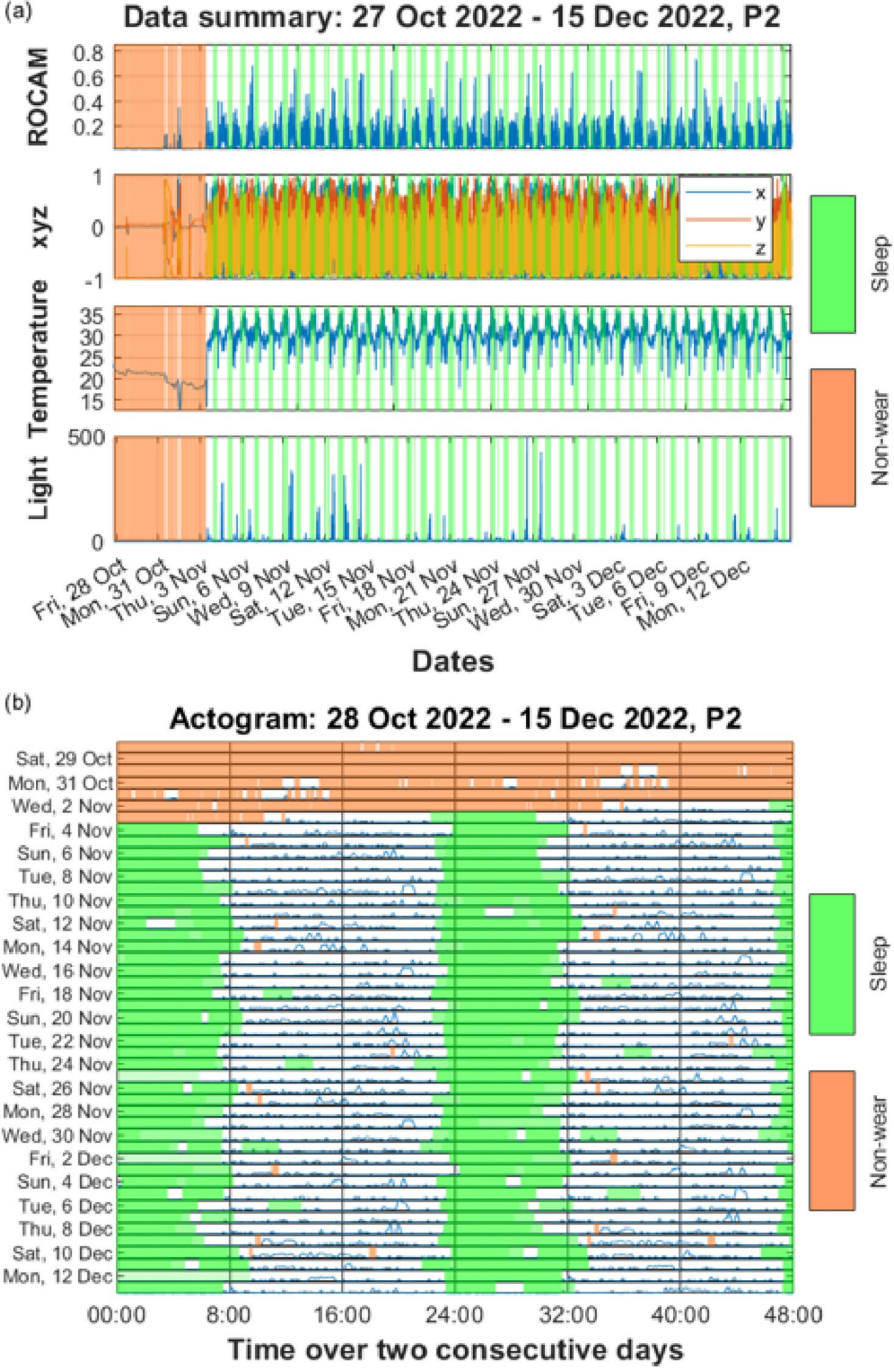

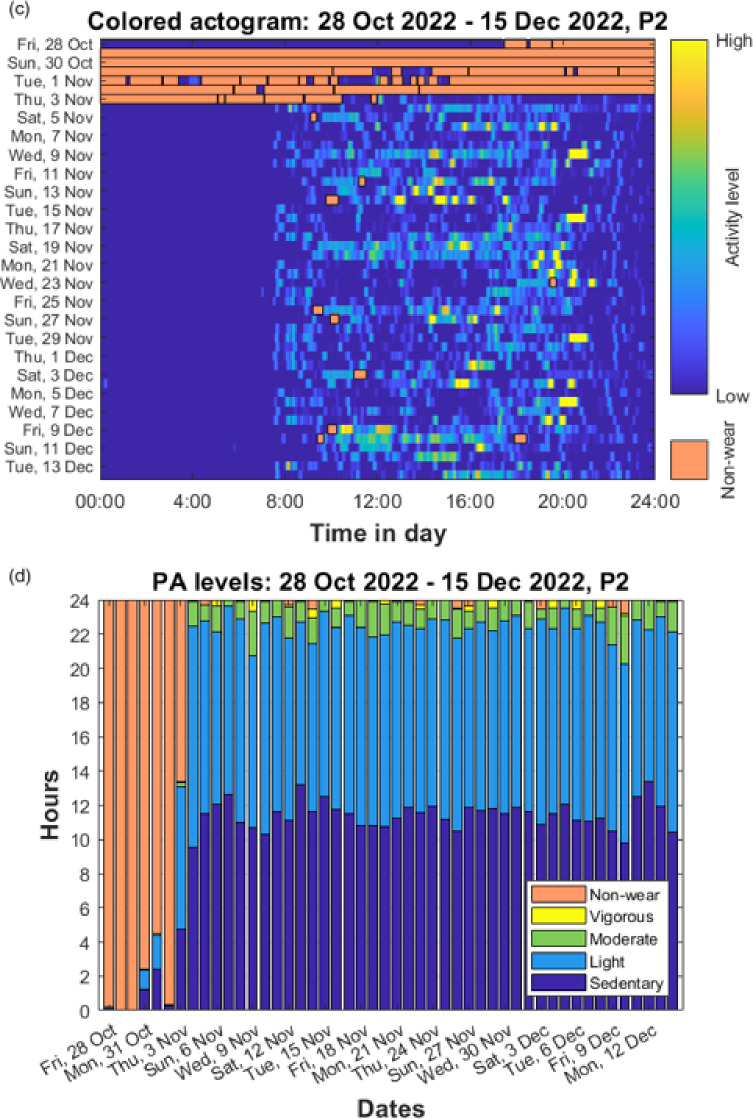

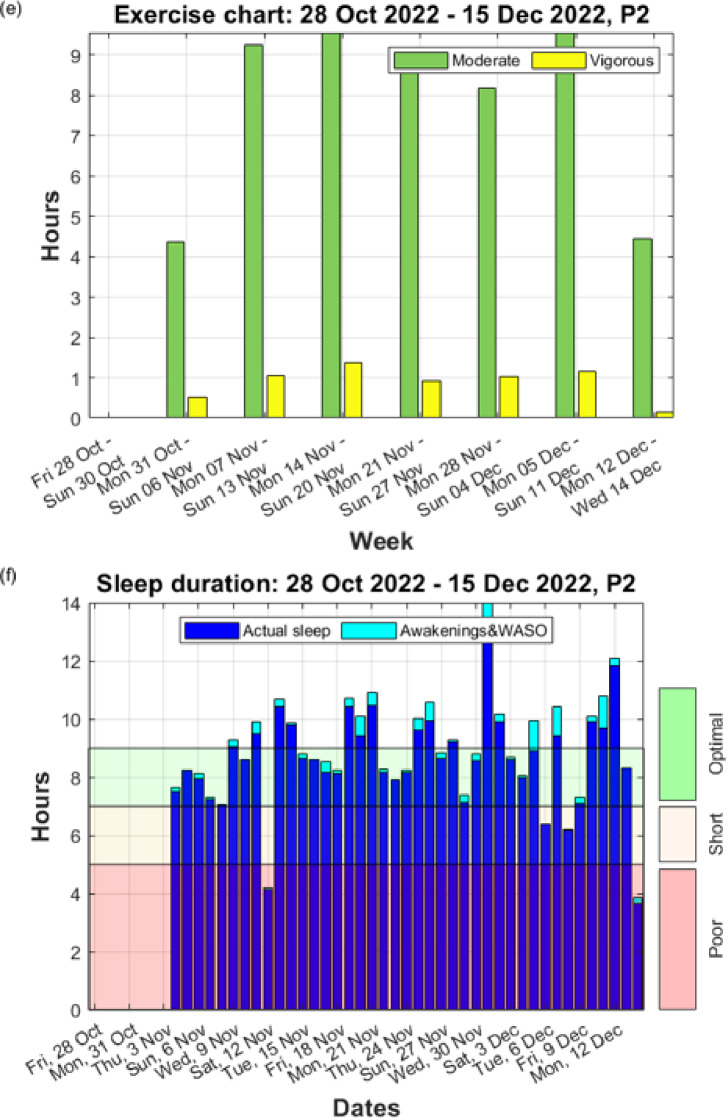

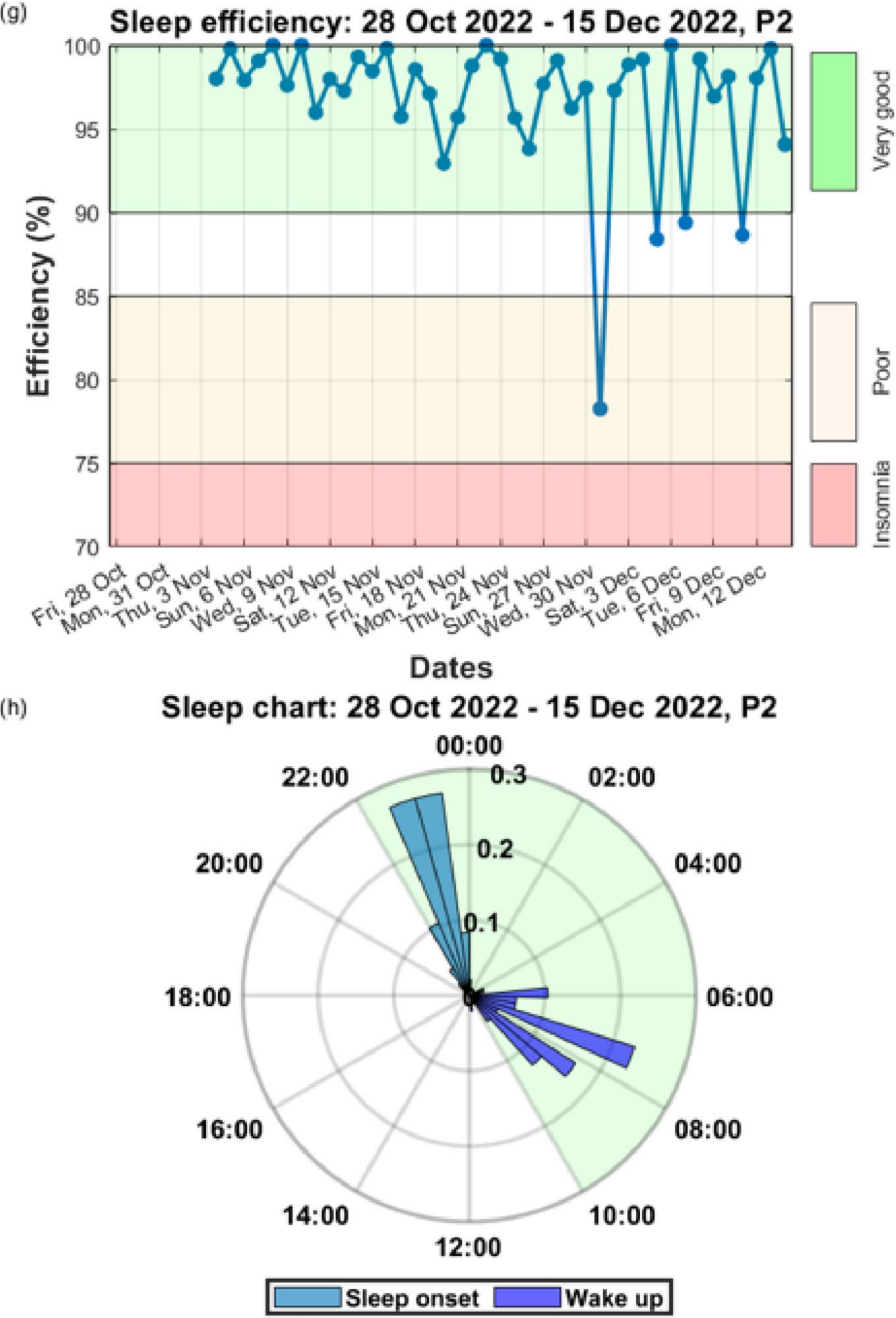
Graphical illustrations of the extracted information from actigraphy data, for the same indicative study participant (P2) as presented in Fig. S3. These personalized graphical outputs are useful to understand a participant’s trajectory, and similar plots were compared when a participant wore the Geneactiv device on multiple occasions.

So far, we have explored the insights that can be gained from processing PROMs at a personalized level for an indicative participant and at the cohort level. Figure 3 presents graphical illustrations of the information that we extracted from the actigraphy data, for the same participant (P2) as presented in Fig. S3. These illustrations, along with the actigraphy measures computed, are used to provide additional continuous, objective, information to complement participants’ inputs via PROMs. Most participants had the wrist-worn wearable sensor on three occasions (see also Fig. 1) so we need to collectively use these plots from the different instances to match against PROMs, or focus on the periods where we have jointly collected both PROMs and actigraphy data.

We can see that participant P2 did not wear the device for the first week or so, presumably the time it took for Geneactiv to reach them (time by the post-office to deliver and collect at their workplace). By visual inspection of the actogram, it is clear this participant had fairly good sleep onset regularity and less good sleep offset regularity (also verified with the sleep chart), and they had some awakenings that were quite prolonged over some nights. The sleep plots suggest that there were no problems with sleep duration, however there were clearly disturbances and awakenings in most nights which affect the participant’s sleep. This is likely what was driving the participant to report poor sleep in general (including the perceived reduced number of hours asleep). The PA plots indicate that the participant engaged in regular weekly PA, matching well what they self-reported in WWQB-B.

**Fig. 4:**
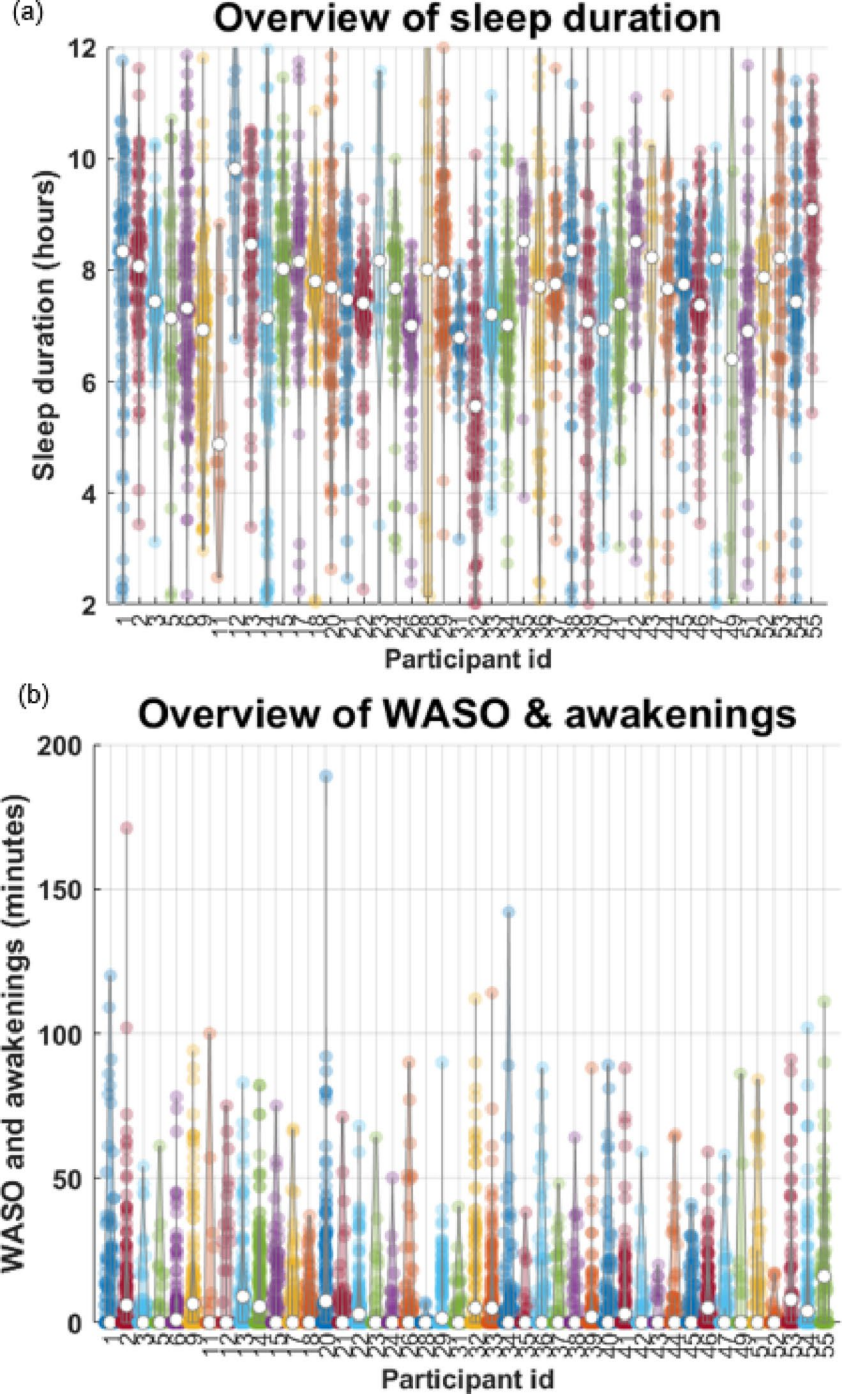

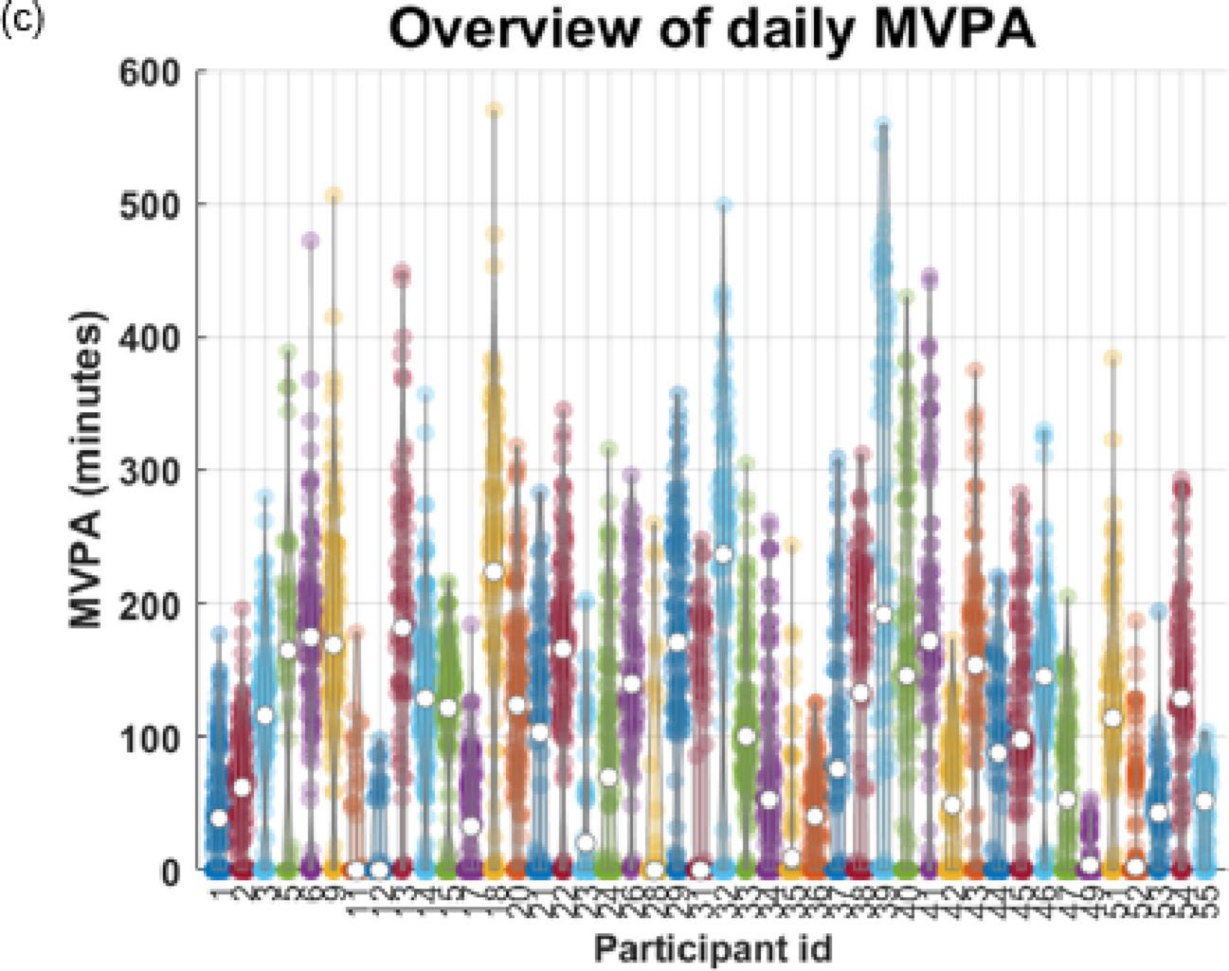
Violin plots providing an overview of indicative actigraphy-extracted characteristics for all participants. We collated the data for each participant for all times they wore the device during the study to provide a succinct overview. The white dot in each violin plot denotes the median, the horizontal line denotes the mean, the transparent vertical line spans from the 25th to the 75th percentile, and the dots are the individual samples. MVPA stands for Moderate-to-Vigorous Physical Activity (the sum of moderate and vigorous activity minutes), and WASO for Wake-After-Sleep-Onset (time spent awake after someone started sleeping, before wake-up to start the day).

Figure 4 presents an overview of sleep duration, WASO and awakenings, and MVPA for each individual participant. We remark that most participants were, in general, sleeping around 7–8 h according to the actigraphy-derived sleep estimates, however almost all have some nights where they clearly had reduced sleep. Moreover, all participants experienced awakenings during their sleep, and for some participants this was the norm. This finding supports the self-reports where participants highlighted sleep problems, sleep disturbances and that they could not sleep well. Many of the study participants engaged in moderate and vigorous daily exercise as estimated from actigraphy analysis, which again reflects well what they had self-reported. Some of the participants did not engage in almost any form of Moderate-to-Vigorous Physical Activity (MVPA), spending the vast majority of their time in sedentary activity (as self-reported in PROMs and verified using actigraphy measures). In Supplementary Fig. S7 we explored whether there are gender-related differences across all these characteristics and found that men, overall, engaged in more daily MVPA and at the same time had lower sleep duration and worse sleep efficiency.

Next, we wanted to gain further insights on the associations between actigraphy measures and PROMs items. Supplementary Fig. S8 illustrates joint actigraphy-PROMs trajectories to visually inspect actigraphy-extracted information and PROMs over the course of the study for an indicative participant. Supplementary Fig. S9 presents the scatter plot of an indicative actigraphy measure (sleep efficiency %) with global PSQI and total WEMWBS to explore the variability of the statistical association for different participants. In this example, the XCF between sleep efficiency and global PSQI (also for total WEMWBS) ranged from approximately -0.65 to approximately 0.65 for different participants. We remark that there were some very strong cross-correlation associations between actigraphy measures and certain PROMs entries for some participants, in some cases denoting starkly opposing relationships (i.e. for some participants indicating proportional relationships, whilst for other participants indicating inversely proportional relationships, as seen in Supplementary Fig. S9). For this reason, the average XCF across all participants was often below the cut-off used here (|*R*| = 0.3).

Table 2 summarizes the key actigraphy measures and their association with PROMs where the average magnitude of the XCFs across participants was large (> = |0.3|), and the insights these provide into participants’ trajectories. Many of these findings are intuitive, for example, people working late (after 22:00) generally had increased nocturnal activity (i.e. movement during sleep which generally indicates worse sleep), and later start of daily activity was generally associated with reduced sleep quality. We clarify that the shortened term ‘night work’ represents WWQB-I item 3, which refers to participants self-reporting on the number of days that they worked between 22:00 and 05:00 over the past month. We had two participants who were shift workers in the study, and six additional participants who self-reported working for at least two hours past 22:00 with some regularity, which was also verified using their actograms (we elaborate on this further in the Discussion).

**Table 2:** Associations of actigraphy measures with PROMs and summary insights.

Actigraphy measure	PROMs entry	XCF	Insights
M10 time	PSQI domain1: quality	0.39 ± 0.23	Later start of the most active 10 h is associated with reduced sleep quality
Sleep offset (wake up)	PSQI domain1: quality	0.32 ± 0.51	Waking up later is associated with reduced sleep quality, although this is quite variable
Sleep temperature offset - zenith time difference	PSQI domain 4: sleep efficiency	0.30 ± 0.36	The difference between the wrist temperature at wake up and max temperature during sleep is associated with reduced sleep efficiency
Sleep activity 50th percentile	PSQI domain 5: sleep disturbances	0.30 ± 0.22	The median movement activity during sleep is associated with increased sleep disturbances
Sleep activity 5th percentile	PSQI domain 6: Medication	-0.36 ± 0.13	Minimal sleep activity (5th percentile) is negatively associated with medication
Sleep activity 25th percentile	WWQB-I item 3: night work	0.33 ± 0.41	People working for more than two hours between 22:00 and 05:00 had on average increased sleep activity
Sleep temperature nadir	WWQB-I item 3: night work	0.31 ± 0.31	People working for more than two hours between 22:00 and 05:00 had on average increased minimum sleep temperature compared to others

For details on the actigraphy measures see the Supplementary Material “Actigraphy_measures.xlsx”. We used ‘PROMs entry’ here to denote active input from participants broadly, including the computed outputs (e.g. PSQI domains) rather than only refer to explicit PROMs items completed by participants. The XCF entries are presented in the form mean±standard deviation. We clarify that the shortened “night work” WWQB-I item refers to people working for at least two hours between 22:00 and 05:00, see also the Questionnaires_in_SHAW.xlsx file for details.

Tables 3, 4 and 5 summarize the results of linear mixed-effects models predicting global PSQI and total WEMWBS using (i) only subsets of PROMs (Table 3), (ii) only actigraphy measures (Table 4), and (iii) a joint subset of PROMs and actigraphy measures (Table 5). In all cases we retained ‘gender’, ‘age’, ‘day into study’ and ‘work sector’ for illustration of their effect on the model outputs. The average intercept was large in comparison to the full scale of both the total WEMWBS and global PSQI scores in all cases, and we also underline its large Confidence Interval (CI): this reflects the considerable underlying differences between participants for the two outcomes explored herein. We remark that specific PROMs items and actigraphy measures were found to be statistically significant predictors of within-person fluctuations, in many cases with relatively large model coefficients (i.e. contribution to the model). The Intra Class Correlation (ICC) when modelling the total WEMWBS was 0.10, 0.39, and 0.32, when using the PROMs-only, actigraphy-only, and joint subset (PROMs and actigraphy), respectively. The ICC when modeling the global PSQI was 0.52, 0.65, and 0.70, when using the PROMs-only, actigraphy-only, and joint subset (PROMs and actigraphy), respectively. Overall, these ICC values highlight that the variables have considerable explanation for the underlying changes in the response (total WEMWBS and global PSQI) for each of the models. The ‘work-life balance’ (quantified via the item WWQBI 10) was prominent in the models as a key predictor, highlighting that this was key for both wellbeing (expressed in WEMWBS) and sleep problems (expressed in PSQI). Additionally, the actigraphy-derived sleep efficiency (in some variant, e.g. using the average or its standard deviation) was consistently one of the statistically significant predictors with relatively large t-statistic scores for both WEMWBS and PSQI. We remark that the presence of both PROMs and actigraphy-derived variables being statistically significant (often with large t-statistic scores) in the model presented in Table 5 demonstrates the complementary effect of actigraphy and PROMs in providing key insights into wellbeing and sleep problems. We defer further elaboration into these findings for the Discussion.

**Table 3:** Results of linear mixed-effects models predicting total WEMWBS and global PSQI using an indicative subset of PROMs.

Y	Variables	Model coef.	95% CI	t-statistic	*p*-value
Total WEMWBS = *f*(PROMs)	(intercept)	50.649	[29.104, 72.195]	4.612	**< 0.001**
	Gender male	− 0.555	[− 2.286, 1.175]	− 0.63	0.529
	Age	0.008	[− 0.369, 0.386]	0.044	0.965
	Day into study	− 0.004	[− 0.006, − 0.002]	− 3.36	**< 0.001**
	Work sector: manufacturing	1.202	[− 2.372, 4.776]	0.66	0.509
	Work sector: social care	− 1.06	[− 4.729, 2.609]	− 0.567	0.571
	Work sector: finance	0.04	[− 2.407, 2.488]	0.032	0.974
	BJSQ 1: much work	0.237	[− 0.179, 0.652]	1.117	0.264
	BJSQ 14: friendly atmosphere	− 0.459	[− 0.769, − 0.149]	− 2.908	**0.004**
	BJSQ 17: worth doing	− 0.408	[− 0.807, − 0.010]	− 2.011	**0.045**
	BJSQ 23: irritable	− 1.591	[− 2.760, − 0.422]	− 2.669	**0.008**
	BJSQ 24: tired	0.897	[− 0.229, 2.023]	1.563	0.118
	BJSQ 27: tense	− 1.468	[− 1.860, − 1.076]	− 7.352	**< 0.001**
	BJSQ 46: can’t sleep	− 0.988	[− 1.344, − 0.632]	− 5.443	**< 0.001**
	WWQBI 1: stressed	0.612	[0.279, 0.945]	3.605	**< 0.001**
	WWQBI 10: work-life balance	1.253	[0.759, 1.746]	4.975	**< 0.001**
Global PSQI = *f*(PROMs)	(intercept)	9.839	[− 1.212, 20.890]	1.747	0.081
	Gender Male	− 0.339	[− 1.202, 0.525]	− 0.770	0.442
	Age	− 0.052	[− 0.245, 0.141]	− 0.532	0.595
	Day into study	0.001	[− 0.000, 0.001]	1.125	0.261
	Work sector: manufacturing	1.938	[0.061, 3.815]	2.026	**0.043**
	Work sector: social care	− 0.547	[− 2.378, 1.284]	− 0.586	0.558
	Work sector: finance	− 1.044	[− 2.279, 0.191]	− 1.658	0.097
	BJSQ 1: much work	0.296	[0.128, 0.465]	3.446	**< 0.001**
	BJSQ 14: friendly atmosphere	0.070	[− 0.055, 0.195]	1.092	0.275
	BJSQ 17: worth doing	0.183	[0.017, 0.350]	2.158	**0.031**
	BJSQ 23: irritable	− 0.455	[− 0.930, 0.020]	− 1.878	0.061
	BJSQ 24: tired	0.651	[0.193, 1.109]	2.789	**0.005**
	BJSQ 27: tense	0.180	[0.021, 0.340]	2.218	**0.027**
	BJSQ 46: can’t sleep	0.525	[0.380, 0.670]	7.116	**< 0.001**
	WWQBI 1: stressed	− 0.198	[− 0.333, − 0.063]	− 2.882	**0.004**
	WWQBI 10: work-life balance	− 0.383	[− 0.584, − 0.181]	− 3.727	**< 0.001**

CI stands for Confidence Interval. See the subsection ‘Linear mixed-effects models’ in the “Methods” section for the generic mixed-effects model formula and further details. The actual variables used in the models are presented in the second column of the Table. We restricted explorations to the work-related questionnaire items to illustrate work-health relationships with total WEMWBS and global PSQI. Bold entries indicate statistical significance at the p = 0.05 level; for convenience we followed the convention that low computed p-values were marked as < .001. See the “Questionnaires_in_SHAW.xlsx” supplementary file for details of each item and its interpretation (e.g. if higher is better). The total WEMWBS lies in the range 14–70, higher being better; the global PSQI lies in the range 0–21, lower being better.

**Table 4:** Results of linear mixed-effects models predicting total WEMWBS and global PSQI using an indicative subset of actigraphy measures.

Y	Variables	Model coef.	95% CI	t-statistic	*p*-value
Total WEMWBS = *f*(actigraphy)	(intercept)	28.407	[− 3.004, 59.818]	1.778	0.076
	Gender_1	− 1.3	[− 3.729, 1.128]	− 1.053	0.293
	Age	0.385	[− 0.168, 0.938]	1.368	0.172
	Day into study	0	[− 0.004, 0.005]	0.205	0.837
	Work sector: manufacturing	0.58	[− 4.605, 5.764]	0.22	0.826
	Work sector: social care	− 1.837	[− 7.007, 3.334]	− 0.698	0.485
	Work sector: finance	− 1.749	[− 5.231, 1.734]	− 0.987	0.324
	Sedentary min_m3	− 0.867	[− 1.467, − 0.266]	− 2.838	**0.005**
	M10 m7	− 12.402	[− 40.863, 16.058]	− 0.857	0.392
	RA m7	23.233	[3.680, 42.786]	2.336	**0.020**
	IS1 m7	13.52	[− 2.100, 29.139]	1.702	0.090
	IV1 m7	7.359	[1.109, 13.609]	2.315	**0.021**
	Sleep duration_m7	− 35.616	[− 61.888, − 9.343]	− 2.665	**0.008**
	Sleep efficiency_m7	− 1.663	[− 3.283, − 0.043]	− 2.018	**0.044**
	Sleep onset phase_v7	0.263	[− 0.285, 0.812]	0.944	0.346
	Awakenings total_minutes_v7	1.214	[0.056, 2.372]	2.062	0.040
	Sleep duration_v7	1.439	[− 6.731, 9.609]	0.346	0.729
	Sleep efficiency_v7	− 5.264	[− 7.979, − 2.549]	− 3.812	**< 0.001**
Global PSQI = *f*(actigraphy)	(intercept)	10.943	[− 2.410, 24.296]	1.612	0.108
	Gender_1	0.133	[− 0.905, 1.171]	0.252	0.801
	Age	− 0.0600	[− 0.293, 0.173]	− 0.508	0.612
	Day into study	0.00100	[− 0.001, 0.003]	0.914	0.362
	Work sector: manufacturing	2.675	[0.423, 4.927]	2.337	**0.020**
	Work sector: social care	− 1.068	[− 3.273, 1.136]	− 0.953	0.341
	Work sector: finance	− 1.451	[− 2.935, 0.032]	− 1.925	0.055
	M10_m3	− 5.76	[− 15.544, 4.025]	− 1.158	0.248
	Sleep duration_m3	6.873	[− 4.808, 18.555]	1.158	0.248
	Sleep efficiency_m3	− 0.357	[− 0.615, − 0.099]	− 2.724	**0.007**
	Sleep onset phase_v3	0.267	[0.074, 0.461]	2.714	**0.007**
	Awakenings total_minutes_v3	0.329	[− 0.227, 0.885]	1.164	0.245
	Sleep duration_v3	1.183	[− 0.546, 2.912]	1.346	0.179
	Sleep efficiency_v3	− 1.508	[− 3.307, 0.290]	− 1.65	0.100

CI stands for Confidence Interval. See the subsection ‘Linear mixed-effects models’ in the “Methods” section for the generic mixed-effects model formula and further details. The actual variables used in the models are presented in the second column of the Table. Bold entries indicate statistical significance at the p = 0.05 level; for convenience we followed the convention that low computed p-values were marked as < .001. See the “Questionnaires_in_SHAW.xlsx” supplementary file for details of each item. The total WEMWBS lies in the range 14–70, higher being better; the global PSQI lies in the range 0–21, lower being better.

**Table 5:** Results of linear mixed-effects models predicting total WEMWBS and global PSQI using an indicative subset of PROMs and actigraphy measures.

Y	Variables	Model coef.	95% CI	t-statistic	*p*-value
Total WEMWBS = *f*(PROMs + actigraphy)	(Intercept)	47.498	[23.614, 71.381]	3.911	**< 0.001**
	Gender_1	− 0.798	[− 2.591, 0.994]	− 0.876	0.382
	Age	0.059	[− 0.360, 0.478]	0.279	0.781
	Day into study	− 0.001	[− 0.006, 0.003]	− 0.626	0.532
	Work sector: manufacturing	1.567	[− 2.486, 5.619]	0.760	0.448
	Work sector: social care	− 0.781	[− 4.622, 3.061]	− 0.400	0.690
	Work sector: finance	− 1.596	[− 4.193, 1.001]	− 1.209	0.227
	BJSQ 23: irritable	− 0.946	[− 1.496, − 0.395]	− 3.376	**< 0.001**
	BJSQ 27: tense	− 0.813	[− 1.437, − 0.189]	− 2.561	**0.011**
	BJSQ 46: can’t sleep	− 0.310	[− 0.861, 0.241]	− 1.105	0.270
	WWQBI 1: stressed	− 0.230	[− 0.705, 0.246]	− 0.95	0.343
	WWQBI 10: work-life balance	2.232	[1.559, 2.904]	6.526	**< 0.001**
	Sedentary minutes_m3	− 0.871	[− 1.399, − 0.344]	− 3.251	**0.001**
	RA_m7	13.876	[4.831, 22.920]	3.017	**0.003**
	IV1_m7	8.487	[2.622, 14.351]	2.846	**0.005**
	Sleep duration_m7	− 24.698	[− 37.67, − 11.725]	− 3.744	**< 0.001**
	Sleep efficiency_m7	− 1.176	[− 2.387, 0.035]	− 1.91	0.057
	Sleep onset phase_v7	− 0.035	[− 0.564, 0.493]	− 0.131	0.896
	Awakenings total minutes_v7	1.052	[− 0.035, 2.139]	1.904	0.058
	Sleep efficiency_v7	− 4.357	[− 6.733, − 1.980]	− 3.605	**< 0.001**
Global PSQI = *f*(PROMs + actigraphy)	(Intercept)	7.057	[− 5.204, 19.318]	1.132	0.258
	Gender_1	0.092	[− 0.852, 1.036]	0.192	0.848
	Age	0.002	[− 0.213, 0.216]	0.0150	0.988
	Day into study	− 0.001	[− 0.003, 0.002]	− 0.580	0.563
	Work sector: manufacturing	2.629	[0.564, 4.695]	2.504	**0.013**
	Work sector: social care	− 1.177	[− 3.197, 0.843]	− 1.147	0.252
	Work sector: finance	− 1.337	[− 2.691, 0.017]	− 1.942	0.053
	BJSQ 1: much work	0.354	[0.022, 0.686]	2.097	**0.037**
	BJSQ 24: tired	− 0.164	[− 0.448, 0.119]	− 1.14	0.255
	BJSQ 27: tense	− 0.155	[− 0.464, 0.154]	− 0.986	0.325
	BJSQ 46: can’t sleep	0.407	[0.127, 0.686]	2.865	**0.004**
	WWQBI 1: stressed	− 0.277	[− 0.508, − 0.045]	− 2.351	**0.019**
	WWQBI 10: work-life balance	− 0.713	[− 1.082, − 0.343]	− 3.796	**< 0.001**
	Awakenings total minutes_m3	0.111	[− 0.077, 0.299]	1.163	0.246
	Sleep duration_m3	− 0.0330	[− 0.437, 0.371]	− 0.162	0.872
	Sleep efficiency_m3	− 0.368	[− 0.605, − 0.131]	− 3.059	**0.002**
	Sleep onset_phase_v3	0.290	[0.098, 0.481]	2.979	**0.003**
	Sleep efficiency_v3	− 0.0560	[− 0.394, 0.281]	− 0.328	0.743

CI stands for Confidence Interval. See the subsection ‘Linear mixed-effects models’ in the “Methods” section for the generic mixed-effects model formula and further details. The actual variables used in the models are presented in the second column of the Table. Bold entries indicate statistical significance at the p = 0.05 level; for convenience we followed the convention that low computed p-values were marked as < .001. See the “Questionnaires_in_SHAW.xlsx” supplementary file for details of each item. The total WEMWBS lies in the range 14–70, higher being better; the global PSQI lies in the range 0–21, lower being better.

Finally, we computed Cronbach’s alpha for the modified (6-point Likert scale) BJSQ using the weekly BJSQ entries. The modified 6-point BJSQ demonstrated excellent internal consistency (alpha = 0.90).

## Discussion

We provided new insights and evidence into the close interrelations of work-related aspects and wellbeing for a closely monitored group of workers aged over 50 using a range of questionnaires and actigraphy-extracted information, over the course of a year. We make several observations where these findings could inform social and healthcare policies, and guide personal choices. First, sleep problems have been consistently self-reported by the majority of participants (global PSQI > 5), strongly corroborated by actigraphy data: these are not due to sleep hygiene (e.g. going to sleep very late or having vastly different sleep patterns in weekends); *they often reflect work-related stress*. Second, overall wellbeing was moderate (total WEMWBS between 43 and 60) and was strongly associated with maintaining a work-life balance and job satisfaction; it was particularly affected when people reported they were gloomy or irritated at their work-place. Third, items pointing to a spectrum of potential mental health problems, such as BJSQ items 30 (“depressed”), 21 (“angry”), and 23 (“irritable”), were associated with work-related aspects, including people reporting feeling tired, lacking energy and not sleeping well.

Some of the key insights obtained from actigraphy data were regarding participants’ sleep patterns and sleep problems, over and above what had been reported via PROMs. Whereas participants often attributed poor sleep (high global PSQI scores) to reduced sleep duration, actigraphy outcomes indicated that it was more often than not due to sleep disturbances and awakenings rather than sleep duration. These disturbances and awakenings were associated with work-related stress, thus creating a plausible causal pathway where work could be affecting sleep and wellbeing. Moreover, through actigraphy processing we could obtain finer resolution on sleep problems on a day-to-day basis (in fact, minute-wise), compared to what was self-reported in monthly PROMs (such as PSQI) and weekly PROMs (BJSQ item 46: “*I haven’t been able to sleep well*”, shortened to “*can’t sleep*” for brevity in the presented plots). Beyond two of the participants who were shift workers, there were six additional participants who self-reported working for at least two hours between 22:00 and 05:00 with some regularity in WWQB-I. Monitoring their actograms, we verified they often went to sleep past midnight, which combined with their PROMs self-response likely reflects work-related pressure/stress (less likely their own working time preference). In turn, working for at least two hours past 22:00 appears to lead to increased sleep activity and increased minimum sleep temperature (see Table 2). Therefore, the presented findings are not skewed by a large proportion of shift work participants. Actigraphy measures matched well, overall, participants’ self-reported outcomes following visual inspection of findings (e.g. looking at individuals’ plots like presented in Fig. 3c, e for PA). Collectively, the findings presented herein further support a large body of research work highlighting the effectiveness of long-term health monitoring through wrist-worn actigraphy for sleep, PA, and diurnal variability assessment^11,33,34^.

Sleep problems (reflected in PSQI and actigraphy) were prevalent across the vast majority of participants in SHAW, particularly for workers in manufacturing (see Fig. 2a); other studies with noncomplaining healthy older adults who were carefully screened had by comparison moderate proportions with self-reported global PSQI > 5 (33% women and 16% men)^35^. This might indicate that the older workforce when not carefully screening out for sleep disorders and mental health conditions might have considerably more sleep-related problems than previously considered (at least in the UK), although this would need to be explored in a larger population sample. Women self-reported more sleep problems compared to men (see Supplementary Figure S2), which is aligned to what has been reported in previous work^35,36^. However, when sleep was measured objectively using actigraphy (see Supplementary Fig. S7) women had somewhat better sleep efficiency with fewer disturbances overall and sleep duration that was more balanced around 8-h sleep on average. These findings further corroborate previous reports on gender comparisons where sleep had been measured objectively using polysomnography^36,37^.

There were some findings from the self-responses across the different questionnaires that might be considered surprising. Participants indicated that sleep (interpreted through the PSQI components) was not highly associated with wellbeing (total WEMWBS score) at baseline, with the exception of the ‘day dysfunction’ component. Similarly, the number of days working longer than 10 hours had little effect on reported work-life balance, when viewed overall across all study participants. Delving deeper and investigating the time series with the month-by-month changes in PSQI and WEMWBS (and similarly week-by-week relationships for other time series), we found this was due to the inherent variability of these relationships across individuals (see Supplementary Fig. S6), hereby highlighting the need to be developing personalized approaches to understand individual trajectories. This was verified using actigraphy too, where the magnitude of some of the XCF relationships between actigraphy measures and PROMs items for certain individuals was very strong (> 0.6, e.g. see Supplementary Fig. S9), however, neither the magnitude nor the direction was consistent across all participants. In a sense, these consistent findings in terms of self-reporting variability and actigraphy measures’ variability across participants underscore the need to understand personalized trajectories and how different work and health aspects are intertwined for each individual.

The linear mixed-effects modeling framework provided an additional layer with more robust inference on the year-long relationships between questionnaire measures and actigraphy-derived variables, allowing for individual participant adjustments. Tables 3, 4 and 5 reinforced XCF findings, pointing to major wellbeing and sleep challenges for the majority of the study participants. The ICC scores were low for WEMWBS and moderate for PSQI. suggesting that the variables in the linear mixed-effects models have considerable explanation for the underlying changes in the responses compared to the intercept (which marks the average response score). Strikingly, poor sleep (as quantified in global PSQI) and wellbeing (as quantified in total WEMWBS) were independent of age and gender, where their effect was almost consistently not statistically significant (*p* > 0.05). In terms of work sectors, working in manufacturing led to overall greater sleep problems in PSQI but did not have an effect on WEMWBS: this is aligned with the baseline PSQI scores presented in Fig. 2a. Work-related aspects (quantified via BJSQ and WWQBI items such as ‘work-life balance’, ‘stressed’ and ‘tense’) had a major impact on people’s sleep and wellbeing. Using the linear mixed-effects models elucidated much better than XCF that challenges participants indicated on BJSQ item 46 (“can’t sleep”) contributed considerably to poor PSQI and WEMWBS outcomes. Interestingly, reporting a lot of work (scored in item BJSQ 1) and being tired (BJSQ item 24) were strongly associated with sleep problems but not wellbeing; whereas having a friendly atmosphere at work (BJSQ 14) was strongly affecting wellbeing but not sleep. We found there were quite different subsets of actigraphy measures that were strongly associated with the two outcomes (total WEMWBS and global PSQI), as reported in Tables 4 and 5. Notably in Table 5, a few of the actigraphy measures were statistically significant with high model coefficient values in the presence of specific PROMs: this underlines they complement self-reports in modeling total WEMBWS and global PSQI. We highlight in particular that the actigraphy-derived sleep efficiency (in its different variants) was a consistently statistically significant predictor of both WEMWBS and PSQI. It is intriguing that it is the standard deviation of sleep efficiency over the last 7 days prior to self-reporting WEMWBS (rather than e.g. the average sleep efficiency) which was identified as a significant predictor: the greater the variability of sleep efficiency, the lower the total WEMWBS. This finding underscores the importance of regular sleep efficiency (i.e. consistently very few awakenings) a concept that is critical in the sleep literature^38^, where arguably actigraphy can be particularly effective. Our experiments indicated that actigraphy measures over the preceding 7 days were most strongly associated with WEMWBS, whereas for PSQI this was over the preceding 3 days prior to self-reporting. This could subtly indicate that participants have a longer memory effect to associate with wellbeing, whereas for sleep problems they focus more on a shorter 3-day span, although this would need to be further validated.

The BJSQ is a standardized widely used tool typically marked on a 4-point Likert scale. In smaller samples or longitudinal studies, a 4-point scale can lead to “clumping”, potentially suppressing small weekly changes due to low granularity. By moving to a 6-point Likert scale in our study, we have effectively increased the sensitivity of the instrument to capture wider variability in responses. However, this modification may alter BJSQ’s psychometric properties and hence the 6-point Likert score setting needed to be validated. Using the weekly BJSQ entries we found that the modified 6-point BJSQ demonstrated excellent internal consistency (Cronbach’s alpha = 0.90). This indicates that the increased granularity of the 6-point scale maintains the psychometric integrity of the original instrument.

When designing the study we carefully considered the number of participants we would aim to recruit bearing in mind that high-frequency reporting (weekly) over a long duration (one year) is a high-burden task. Arguably, for the purposes of monitoring longitudinal trajectories it is preferable to have 50 highly engaged participants with a 60–80% completion rate than 200 participants with a 30% completion rate. The determination of the required sample size was motivated by prior empirical work suggesting that about 50 participants may often be sufficient in longitudinal studies^39^. Previous simulation work had suggested the empirical 30/30 rule (30 participants and 30 entries per participant, the latter sometimes referred to as “repeated measures” or “intensive longitudinal data”) whereas more recently the 50/20 rule has been suggested as a useful heuristic (50 participants and 20 entries per participant) for longitudinal studies^40^. The conventional power analysis computation using the power analysis formula (see the corresponding section in the Supplementary Material entitled ‘Power analysis’)^41^, yielded a sample size of 32 participants given some conservative assumptions. Our original sample size of 55 participants was set to comfortably meet these recommendations, accounting for some potential drop-outs. The resulting size of *N* = 45 participants meets the power analysis estimates and is broadly aligned with minimum empirical sample size recommendations comprising a high number of Level-1 observations (*n* for both PROMs and actigraphy) at a slightly reduced number of participants *N* (when using the 50/20 empirical rule as guidance). There is a large number of established self-reporting instruments in the literature and there is an inherent trade-off and consideration to select the appropriate instrument(s) depending on the needs of a study and participant engagement/feedback^42^. We chose to use well-established questionnaires capturing work-related aspects (BJSQ, WWQB), general wellbeing (WEMWBS), and sleep (PSQI) to get a broad understanding into participants’ lives. We remark that these choices entailing time commitments considerations, were made through co-design with participants before trial onset to ensure that participant burden was reasonable towards maximizing retention and adherence rates. Most remote studies focusing on digital health are relatively short, whilst retention rates and adherence rates vary widely^43,44^. There is no objective or agreed definition to characterize retention rates and adherence rates as “good” or “satisfactory” since this depends on the duration and nature of the study: indicatively, PROMs completion rates in previous studies varied between 41% and 91.5%^44^. The retention rate, overall PROMs adherence and actigraphy adherence (~ 67%) in this longitudinal study are on the higher end of those previously reported in the literature and hence may be considered fairly satisfactory. This subjective assessment is anchored on that this was not a clinical group, some participants may have not been strongly incentivized to participate longitudinally (e.g. through payment or other incentives), and we did not have a dedicated team tasked with regular follow-up of participants. To the best of our knowledge, the only other longitudinal study (over one year) in the digital health literature that required regular active input and passively collected sensor-based data which reported considerably better adherence (~ 80%) was the Activity Monitoring of Mood Symptoms (AMoSS) study^13,15,16^. However, AMoSS was considerably better resourced than SHAW and there were subtle differences improving retention as outlined in recent work^43^: (i) a dedicated nursing team regularly following up participants via phone calls, (ii) participants were clinical groups and a control group many of whom had specific interest in the study’s objectives (e.g. because of a loved one having a clinical diagnosis in this space), (iii) participants were strongly incentivized to participate (through payment of monthly phone bills and the retention of what was at the time a state-of-art Android smartphone if they provided data over a year).

The Geneactiv wrist-worn wearable sensors used in the study do not provide a portal for participants to view their own data: as part of the co-design we pledged that at the end of the study participants would receive personalized reports with their PROMs, summary graphs of their actigraphy data, and explanations of what these mean in plain language. To avoid any biasing during the data collection phase, we delivered those reports after the study concluded. Participants reported high satisfaction with the personalized feedback. Anecdotal evidence suggests that earlier transparency regarding the value of the inferred data could have further improved sensor adherence. To improve retention in longitudinal actigraphy studies, we recommend engaging participants early through co-design to encourage them become active stakeholders. Providing personalized end-of-study reports further incentivizes participation; by demonstrating how consistent wear-time directly enhances the precision of their own health insights, participants become more intentional about maintaining data quality.

There are many techniques in the research literature developed across different disciplines to assess the similarity (or dissimilarity) between two or more time series^45–49^. Many of these techniques rely on long time series (e.g. preferably thousands of samples), and often regularly sampled time series (i.e. sampled at regular, specific time intervals) and hence they are not applicable for the data in this study. Therefore, we have been guided by practical limitations on the choice of algorithmic tools we could use to process the time series available here. We emphasize that the use of these tools here was to guide our considerations for exploring strong associations between time series, before we looked at person-specific pathways to understand what these relationships might indicate. Similarly, this was a guiding principle when designing the linear mixed-effects models where we needed to decide on a small set of variables to jointly explore due to the practical problems (collinearity) pertinent when using larger number of variables that may be highly correlated (see the heatmap expressing PROMs correlations in Fig. S4, for example).

The growing population of workers over 50 are vital to their workplace, their family, and wider society, many adapting their employment and personal circumstances to attend others’ needs, including providing childcare and looking after older relatives^50^. Given that their healthcare needs are bound to be increasing in mid-to-later life leading up to retirement and beyond due to aging^1,2^, there is collective effort needed to improve long-term outcomes. Although it is critical that individuals embrace World Health Organization (WHO) guidelines and national recommendations promoting active aging such as sleep hygiene and regular weekly PA^51^, similarly, appropriate frameworks and policies should be further reinforced at workplaces to facilitate employer support towards individuals’ wellbeing^52^.

As healthcare models increasingly transition to digital environments, contemporary research underscores a fundamental move toward proactive care and online delivery^53–55^, also reflected in the 2025/2026 priorities and operational planning of the National Health Service (NHS) (https://www.england.nhs.uk/long-read/2025-26-priorities-and-operational-planning-guidance/). The ambition for online digital healthcare delivery is achieved through long-term, personalized monitoring with continuous, data-driven insights, integrated in individuals’ daily life. Arguably, passively collected information through digital technologies which are easy to use (such as wrist-worn wearables) may be a crucial component of this endeavour, complemented by soliciting PROMs (*health-related* and *work-related*). We envisage the proposed data analysis pipeline reported herein (comprising PROMs and information extracted from the wrist-worn sensor data) could be integrated in a deployable platform which could serve: (i) to empower individuals understand their own health trajectories; (ii) to provide healthcare experts a more holistic framework for personalized healthcare overview, possibly used in clinical consultations with an individual; and (iii) to enable policy-makers better monitor health-work related outcomes. It is conceivable this pipeline could be integrated as part of the widely used NHS app in the UK (https://digital.nhs.uk/data-and-information/publications/statistical/nhs-app-statistics), which is transitioning from a simple tool for viewing records into the primary “digital front door” for the NHS.

The primary limitation of the study is the relatively small number of participants, suggesting that findings should be carefully interpreted, particularly in terms of how well they generalize across different work sectors. Nonetheless, there was good internal consistency across the four work sectors explored here and some very strong associations reported, which provides reasonable confidence in the interpretation of findings. To our knowledge, there are no other large longitudinal detailed studies which combine self-reporting and actigraphy to provide insights into the intersection of work aspects and wellbeing. We also remark that the sample size used herein is comparable to other studies collecting raw actigraphy data longitudinally, e.g. Hachenberger et al.^56^ collected data from 50 participants for about 2 weeks and Thurman et al.^57^ collected data from 30 participants for up to 112 days. A secondary limitation is that we did not use tailored questionnaires to capture mental health symptoms, and relied on specific items within the broader questionnaires (such as BJSQ) and WEMWBS to infer such problems. Similarly, participants were not physically assessed by a medical expert at any stage of recruitment and we did not explicitly aim to exclude people with potential chronic or acute pathologies, which may be considerably affecting their wellbeing. Also, to the authors’ knowledge the BJSQ has not been previously validated in the UK setting, and changing the scale format (from the validated 4-point Likert scale to a 6-point Likert scale in this study) means the reported BJSQ results herein are not directly comparable to other studies. Finally, we acknowledge that in order to portray overall health and wellbeing trajectories more holistically, it would be useful to link the existing data to individuals’ EHRs: future work should likely explore how different sources of health information can be optimally considered *jointly*.

Remote health monitoring in older adults combining *actively* collected data in the form of self-reports and *passively* collected data using digital technologies, followed by advanced data processing will likely shape the future of healthy aging^22,58^. We demonstrated that integrating information extracted from work-related self-reports and wrist-worn actigraphy can provide useful insights into older workers’ wellbeing and health, and should likely be embedded into future smart-health monitoring systems.

## Methods

### Study background and setup

The Supporting Healthy Ageing at Work (SHAW) project was conceived to support the health and wellbeing of older workers (50+ years). We worked with employers, employees, professional bodies, and other key stakeholders, on deepening the understanding of how work and health are intertwined in older workers’ everyday lives. For that reason, participants were recruited with the aim to collect data for up to a year to obtain a longitudinal overview of their trajectory. To obtain an indicative perspective of how different professions might impact on wellbeing, we collected data from participants across four sectors: (i) manufacturing, (ii) social care, (iii) finance, and (iv) self-employed. For the first three sectors we approached specific organizations, whereas the last sector is a group of self-employed individuals. These sectors were selected to have indicative workforce representation and diversity, taking into account practicality considerations (employer buy-in and contacts to support employee engagement in the study). The inclusion criteria were: people at the later stages of working life (defined as above 50 years of age), working (employed or self-employed) at the time of recruitment, willing to wear a wrist-worn wearable sensor and self-report on questionnaires in a year-long study. We did not explicitly apply exclusion criteria beyond the above requirements (e.g. excluding shift workers or on the basis of physical and mental health problems).

We recruited 55 participants aged 50–66 years old, seven of whom withdrew prior to the study onset and three withdrew during the study due to unforeseen work and/or health changes. For the power analysis considerations to decide on that sample size please see the section ‘Power analysis’ in the Supplementary Material. The 45 SHAW participants worked in manufacturing (*n* = 7, 59.00 ± 5.51 years, 6 male), social care (n = 5, 55.80 ± 4.66 years, 2 male), finance (*n* = 26, 56.42 ± 4.13 years, 10 males), and in self-employment (n = 7, 56.25 ± 5.38 years, 2 male). Participants were recruited through emails and posters within the three organizations that collaborated within the wider project with the research team, and a non-profit organization supporting self-employed workers. Participation in the study was anonymous to employers, with the explicit agreement in advance of recruitment that participants could participate in the SHAW study during their normal working hours. The study received ethical approval from the Research Ethics and Integrity Review Group (REIRG), University of Edinburgh, CAHSS2107/04, ID: 3186, Title: 6f57-c21c-fa44-34e9. All research was performed in accordance with relevant guidelines/regulations and in accordance with the Declaration of Helsinki. Participants provided informed consent upon recruitment and could withdraw participation in the SHAW study at any point, for any reason, without needing to provide justification to the research team.

We collected four types of data modalities: (a) voice, in the form of interviews with a member of the SHAW team (at the study baseline with all participants, and at the end of the study for a selected number of participants); (b) baseline PROMs, weekly PROMs, and monthly PROMs, using established, validated questionnaires; (c) actigraphy data using the Geneactiv wearable building on the experience and expertise we have using that device in related studies^24,30,31^, and (d) an option to provide some comments (free text) alongside PROMs. We designed the study so that the weekly and monthly self-reports were complementary: in some weeks participants were encouraged to register both a weekly entry and a monthly entry. We elaborate further below in detail on the PROMs and the actigraphy data collected, which are the focus of the present study. The information from the interviews will be reported separately in a follow up study. The free text option was rarely used, e.g. participants indicating they would go on holidays.

**Fig. 5:**
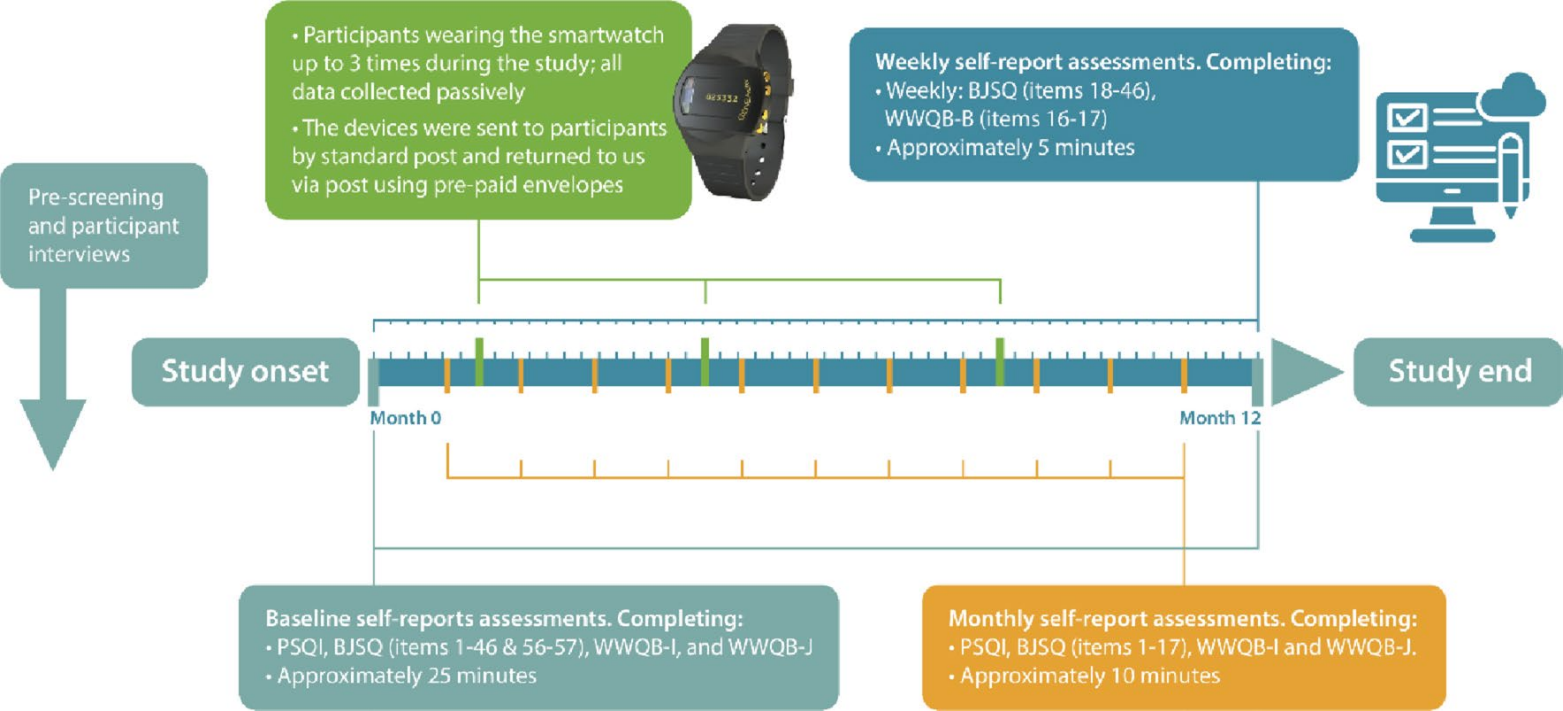
Participant journey in SHAW including all the questionnaires used (requiring *active* input from a participant) and the data collection with the wrist-worn wearable (*passively* collected data, i.e. a participant does not need to do anything specific other than wearing the watch and continuing as usual on their normal activities). Participants were requested to wear the wrist-worn wearable for about 40 days each time (three times in total during the study period).

An overview of the participant journey in the SHAW study is summarized in Fig. 5. In total, the *active* participant burden was about 25 minutes for baseline PROMs, about 5 minutes for weekly PROMs, and about 10 minutes for monthly PROMs.

### Questionnaires in SHAW (self-collected PROMs)

We used a range of job-related and wellbeing-related established questionnaires to assess longitudinal outcomes. In all cases we collected PROMs using the Research Electronic Data Capture (REDCap) tool^59^, which was configured so that prompts were sent to elicit participants’ weekly and monthly responses. Our key considerations were to: (a) use complementary established questionnaires to assess work-related and health-related outcomes, and (b) keep participant burden low, where all entries could be completed within a few minutes. The data was stored directly in secure University of Edinburgh servers at the back-end of our REDCap database. Below we summarize details of the questionnaires collected, and the frequency with which these were collected in SHAW. For easier reference and completeness, we also include as Supplementary Material an Excel file summarizing all items of each questionnaire (see “Questionnaires_in_SHAW.xlsx”).

#### Pittsburgh Sleep Quality Index (PSQI)

PSQI is a self-report sleep assessment tool comprised of 19 items^60^, which has been extensively used in sleep studies^38^. Most of the PSQI items are in the form of a 4-point Likert scale (some other items in the form of hours of sleep). Following standard PSQI guidelines^60^, these items were subsequently mapped onto seven domains: (i) subjective sleep quality, (ii) sleep latency, (iii) sleep duration, (iv) habitual sleep efficiency, (v) sleep disturbances, (vi) use of sleeping medication, and (vii) daytime dysfunction. Each domain was scored in the range 0–3 (0 indicating no symptoms), and the sum of the seven domain scores generated the total score known as *total PSQI* (or *global PSQI*), which has a range 0 to 21: scoring above 5 is used as a typical threshold to indicate poor sleep^60^.

PSQI was collected at baseline and monthly, with the timeframe referred to self-reporting over the last month.

#### Brief Job Stress Questionnaire (BJSQ)

BJSQ has been widely used in occupational health studies and practice to assess psychosocial factors and stress at work for occupational safety and health management^61^. Conceptually, BJSQ proposes criteria to define high-stress workers and has been used as part of a stress check program piloted towards screening high-risk workers and supporting them to consult with a physician. It consists of 57 items which can be broadly considered to cover the following domains: (i) job stressors (17 items), (ii) psychological and physical stress reactions (29 items), (iii) support for workers (9 items), and job and life satisfaction (2 items). Items 1–17 were scored in a 4-point Likert scale, and items 18–46 in a 6-point Likert scale to capture wider variability (the currently validated BJSQ version uses a 4-point Likert scale throughout for all items). BJSQ needs to be interpreted carefully on item-by item basis: for some items higher scores are better, whilst for other items lower scores are better. We stress that because we used a different response format (with 6 levels instead of 4), the outputs for this questionnaire cannot be directly compared against other studies which might offer e.g. normative data.

BJSQ items 1–46 and 56–57 were collected at baseline (the timeframe for these was over the past month); BJSQ items 18–46 were collected weekly; and BJSQ items 1–17 were collected monthly. The choice of the items collected at different times was on the basis of our experience, initial participant involvement during co-design, and the pragmatic consideration of trying not to overburden participants. Inherently in the choice of the regularity of soliciting responses for specific items we had factored in how frequently changes might be expected at different time-scales e.g. weekly or monthly. The timeframe for the recurring collection of the BJSQ items 18–46 referred to self-reporting over the last week, and items 1–17 over the last month.

#### Warwick–Edinburgh Mental Wellbeing Scale (WEMWBS)

WEMWBS was developed to assess mental wellbeing and has been used in many different settings, including different workplaces, and UK population statistics are available for comparisons^62^. All items are worded positively, covering feeling and functioning aspects of mental wellbeing, thereby making the concept accessible for wide use in the general population. WEMWBS comprises 14 items scored on a 5-point Likert scale (1…5, with 1 indicating the least satisfactory response). The sum of these items gives rises to what we refer as *total WEMWBS*, which lies in the range 14–70: higher score indicates better wellbeing. The interpretation is that wellbeing is: (i) low when the score is less than 43, (ii) moderate when the score is 43–60, and (iii) high when the score is higher than 60^62,63^. For further context and an up-to-date platform for this questionnaire see (https://warwick.ac.uk/fac/sci/med/research/platform/wemwbs).

WEMWBS was collected at baseline and monthly, with the timeframe referred to self-reporting over the last month.

#### Workplace Wellbeing Question Bank (WWQB)

WWQB is a large item bank of validated questions, designed to cover all key aspects of wellbeing derived from existing frameworks of wellbeing and work, aiming to measure and monitor the wellbeing of employees^64^. Conceptually, WWQB is similar to the Patient-Reported Outcomes Measurement Information System (PROMIS) item bank, which has been extensively used to elicit self-reporting health-related outcomes^65^. Like PROMIS, WWQB provides a flexible framework to choose and adapt items for the particular needs of a study. Items are scored mostly in a Likert scale (some items with three possible outcomes: “yes”, “no”, “don’t know”) with lower values denoting the least satisfactory response for that item (e.g. “never” or “not at all”, depending on the question). WWQB is organized around 11 main themes (item pools) denoted by A…K (for details, see: https://whatworkswellbeing.org/wp-content/uploads/2020/07/question-bank-workplace-wellbeing-July2020.pdf). We summarize below the WWQB themes that were used (or adapted) for the needs of our study.

WWQB-B (Health outcomes and behaviors) comprises 20 items. Here, we used items 1–4 and 17–18 (the latter two capturing physical activity aspects). We used the entire WWQB-I (Working time quality, 10 items) and the entire WWQB-J (Physical Environment, 4 items). We made a slight adaptation to the third item of WWQB-J adding the following explanatory comment: “*Interpret the term tools as relevant to your job*.”

In terms of WWQB we collected: (a) baseline: WWQB-B, WWQB-I, and WWQB-J; (b) weekly: items 17–18 of WWQB-B; (c) monthly: WWQB-I and WWQB-J. The timeframe for the baseline collection referred to over the last month. The timeframe for the recurring collection referred to self-reporting over the last week for items 17–18 in WWQB-B, and over the last month for WWQB-I and WWQB-J.

### Actigraphy data

We used the Geneactiv Original (https://activinsights.com/technology/geneactiv/) wrist-worn wearable which collects three data modalities: (a) raw three-dimensional acceleration, (b) ambient light, and (c) wrist temperature. The dynamic range for the acceleration data is set by the manufacturer: ±8 g (“g” is a gravity unit, 1 g = 9.81 m/second^2^) and 12-bit resolution. The sample rate is configurable at 10, 25, 50, or 100 Hz, with an intrinsic trade-off where higher sample rate leads to fewer days of data collection. We set the sample rate to 10 Hz to maximize longitudinal data collection (about 40–45 days on a single charge), building on previous work this is fully sufficient for day-to-day PA and sleep assessments^25^^34^.

The Geneactiv devices were posted to participants using the standard UK Royal mail service and included pre-paid envelopes for their return to the research team. Following initial consultation with our public involvement group, we requested participants wore the devices up to 3 times spread throughout the year. We asked participants to wear the device for about 40 days each time, to maximize the data that can be captured on a single charge. Strategically, we elicited PROMs responses at least 2 weeks before participants were sent the wearable devices for the first time, so that we have a baseline of self-reports before collecting concurrent PROMs and wearable data.

### Assessing adherence

Given the longitudinal nature of data collection in SHAW including *active input* (PROMs) and *passive input* (wearables), it is important to assess overall adherence (i.e. participants engaging to provide data). For the PROMs we need to account for some slight variability in when participants completed the questionnaires. We used a simple strategy that was employed in related studies^13,15^: a participant was considered to have been adherent for a particular entry in weekly PROMs if their entry was successfully completed within two days and for monthly PROMs within six days from the expected completion date; otherwise, that entry was marked as missing.

For the wearable data we considered that a participant was adherent for a particular day and provided “valid” data when they wore the device for at least 20 hours during a 24-hour day (standard calendar day). Deciding whether a particular day should be processed or discarded is particularly important to obtain an objective overview of day-to-day variability in terms of PA, sleep, and diurnal variability patterns, whilst allowing for some time that participants may have removed the devices.

### Processing the raw actigraphy data

Typically, the raw three-dimensional acceleration data is summarized using an *acceleration summary approach* to bring into vector format^34,66^. We used the recently proposed Rate of Change Acceleration Movement (ROCAM) to summarize the triaxial signal, which was defined as^34^:

1 $$\:{R}_{i}\stackrel{\scriptscriptstyle\mathrm{def}}{=}\sqrt{{\left[{x}_{i}-{x}_{i-1}\right]}^{2}+{\left[{y}_{i}-{y}_{i-1}\right]}^{2}+{\left[{z}_{i}-{z}_{i-1}\right]}^{2}}$$

where *\:x,y,z* refer to the three-dimensional acceleration axes.

ROCAM intuitively overcomes some shortcomings of competing acceleration summary measures due to the inherent properties of the derivatives across each axis, and was shown to more accurately capture the underlying sleep and PA characteristics than competing approaches (see^34^.

Before further analysis of the actigraphy data, we first computed Non-Wear Times (NWT) to identify segments which should be excluded from analysis. When there was more than 4 NWT hours in a single day, we discarded that day from further analysis. NWT estimation was achieved by a combination of detecting comparatively low temperature and low acceleration variation for periods lasting at least 15 minutes following previous work^24,67^. We used the NWT algorithm we had previously proposed^24^.

### Inferring sleep from actigraphy data

Sleep onset and offset were computed by further processing ROCAM using the algorithm we previously validated^24^. This algorithm had been shown to be more accurate than widely used competing approaches for sleep estimation, e.g. the algorithm in the GGIR package^68,69^. In brief, our sleep estimation algorithm first computes sleep candidate minute-by-minute entries using empirical ROCAM thresholds. Subsequently, we applied a smoothing step to determine whether sleep candidate segments with a duration of at least two hours can be labeled as sleep. For further details, see^24^.

#### Actigraphy measures

We extracted 55 sleep, PA, and diurnal variability characteristics (collectively referred to as *actigraphy measures*) largely building on our previous work^24,30,34^. We clarify that this categorization into three broad types is not unique and is used for convenience in presentation and grouping. Each of the actigraphy measures was computed for each day, typically resulting in ~ 35 × 55 (days×actigraphy measures) matrix every time participants wore the device.

Specifically, we computed: *M10* as the average ROCAM for the 10 most active consecutive hours in a 24-hour day using minute-wise assessments, as described in Blume et al.^70^, *M10 time* marking the start of the 10 most active hours^24^; *L5* denoting the average ROCAM for the least 5 active hours using minute-wise assessments^70^, *L5 time* marking the start of the 5 least active consecutive hours^24^; and the *Relative Amplitude* (*RA*) which measures the relative difference between the most active hours and least active hours:

2 $$\:RA\:=\left(M10-L5\right)/\left(M10+L5\right)$$

We used the methodology we reported on previously applying ROCAM thresholds^34^ in the period during the day which is not labeled as sleep (as defined in the preceding section) to estimate the standard PA categories: *sedentary*,* light*,* moderate*,* and vigorous activity*. To conform with PA guidelines and the actigraphy literature we also computed the *MVPA* which is simply the sum of moderate and vigorous activity. In all cases, this refers to minutes per day in each of these PA categories.

We also computed the *Mean Diurnal Activity (MDA)*, as the average ROCAM during wake-times; *Mean Nocturnal Activity (MNA)*, as the average ROCAM during sleep, and the *Mean Activity (MA)*, weighting diurnal and nocturnal components as in Faedda et al.^71^:

3 $$\:MA\:=\frac{{R}_{nocturnal}\bullet\:{t}_{nocturnal}+{{R}_{diurnal}\bullet\:t}_{diurnal}}{{t}_{nocturnal}+{t}_{diurnal}}$$

where $\:{R}_{nocturnal}$ is the averaged 5-min ROCAM epochs during sleep, $\:{t}_{nocturnal}$ is the sleep duration, $\:{R}_{diurnal}$ is the averaged 5-min ROCAM epochs during wake time, $\:{t}_{diurnal}$ is the total time not spent in sleep (times in all cases in minutes).

Conceptually similar to MA, is the *percent nocturnal activity (%NA)*, which was computed as the ratio of average nocturnal ROCAM over the total average ROCAM per day. A further set of measures we extracted was *percentiles (5*,*25*,*50*,*75*,*95) of ROCAM from wake-up to bed time* assessing overall diurnal activity, and the *Time Dependent Coefficient of Variation (TD-COV)*, measuring the ROCAM variability across the diurnal epochs $\:{N}_{d}$ marking the wake-up period(s):

4 $$\:TD-COV\:=\frac{\frac{1}{{N}_{d}-1}\bullet\:\sum\:_{i=2}^{{N}_{d}}{\left({R}_{i}-{R}_{i-1}\right)}^{2}}{\frac{1}{{N}_{d}}\bullet\:\sum\:_{i=1}^{{N}_{d}}{\left({R}_{i}\right)}^{2}}$$

Subsequently, we used two generic vector operators to process data: the Teager–Kaiser Energy Operator (TKEO)^72^, and the Root Mean Squared Successive Differences (RMSSD):

5 $$\:\mathrm{T}\mathrm{K}\mathrm{E}\mathrm{O}\:=\frac{1}{{N}_{d}-2}\sum\:_{i=2}^{{N}_{d}-1}\left({{R}_{i}^{2}-R}_{i-1}{\bullet\:R}_{i+1}\right)$$

6 $$\:\mathrm{R}\mathrm{M}\mathrm{S}\mathrm{S}\mathrm{D}\:=\sqrt{\frac{1}{{N}_{d}-1}\left(\sum\:_{i=1}^{{N}_{d}-1}{\left({R}_{i+1}-{R}_{i}\right)}^{2}\right)}$$

In addition, we computed the *activity ratio TKEO*, defined as the ratio of the average TKEO activity during the diurnal time over the sum of the average TKEO activity during diurnal time and nocturnal time. Similarly, we computed the *activity ratio RMSSD*, which has an identical definition except for using RMSSD rather than TKEO, as defined in^24^.

From the estimation of sleep (see preceding section) we also computed the following which were used as actigraphy measures: *sleep onset*, *sleep offset* (wake-up time), *sleep duration*, the sleep activity *percentiles (5*,*25*,*50*,*75*,*95)* which indicate ‘activity’ during the period denoted as sleep, and *sleep entropy* to quantify the variability of activity during sleep.

Focusing on the short segments marking sleep interruptions during the period estimated as night sleep, we also computed the *number of awakenings* per night and the *total number of minutes that awakenings lasted* per night (typically short durations, < 5–10 min). Similarly, for longer durations of periods someone was awake in intermittent periods during time in bed we defined Wake-After-Sleep-Onset (WASO), which typically are longer in duration (> 10 min) recording both the *number of WASO* and *total WASO duration*^24^. We computed the *sleep efficiency* as the percentage of time spent sleeping over total time in bed (time asleep, WASO times and awakenings times). Furthermore, we computed differences between successive days in terms of sleep onset timing (*sleep onset phase*) and sleep offset timing (*sleep offset phase*).

Next, we computed the Inter-day Stability (IS), which expresses the stability of activity across days (lies in the range 0 to 1, with values close to 1 indicating strong coupling with external zeitgebers such as sunlight):

7 $$\:IS\:=\frac{\frac{1}{k}\bullet\:\sum\:_{h=1}^{k}{\left({\stackrel{-}{R}}_{h}-\stackrel{-}{R}\right)}^{2}}{\frac{1}{1440}\bullet\:\sum\:_{i=1}^{1440}{\left({R}_{i}-\stackrel{-}{R}\right)}^{2}}$$

where $\:{\stackrel{-}{R}}_{h}$ is the mean activity sampled over *\:k* instances, $\:{R}_{i}$ is the activity at the *\:i*th minute, and $\:\stackrel{-}{R}$ is the overall average activity. We computed two IS variants: using averages over 1 hour (*IS1*), and over 1 hour with a 30-minute overlap (*IS2*).

Additionally, Intra-day Variability (IV), is complementary to IS and quantifies the fragmentation of the diurnal rhythm:

8 $$\:IV\:=\frac{n\bullet\:\sum\:_{i=2}^{n}{\left({R}_{i}-{R}_{i-1}\right)}^{2}}{\left(n-1\right)\bullet\:\sum\:_{i=1}^{n}{\left({R}_{i}-\stackrel{-}{R}\right)}^{2}}$$

where *\:n* is the number of samples used to analyse the variability, giving rise to three IV variants: we used *\:n=1440* (minute-wise summarized activity, *IV1*), *\:n=24* (1-hour summarized activity, *IV2*), and *\:n=48* (1-hour summarized activity with a 30-minute window overlap, *IV3*).

Finally, building on recent work demonstrating that wrist-temperature provides additional information over and above the processing of acceleration signals towards diurnal variability assessment in human studies^30^, we extracted the following: the *sleep temperature zenith* (maximum temperature during sleep), *sleep temperature zenith time* (time of maximum temperature during sleep), *sleep temperature nadir* (minimum temperature during sleep), *sleep temperature zenith time* (time of minimum temperature during sleep), and *sleep temperature range*. These were complemented with the timing differences between temperature at sleep onset with zenith time, temperature onset with nadir time, temperature offset with zenith time, and temperature offset with nadir time.

For easier reference we have summarized, categorized, and provided succinct descriptions for all the computed actigraphy measures in the Supplementary Material titled “Actigraphy_measures.xlsx”. To explore aligning each of these actigraphy measures with PROMs we have also computed summary measures (mean and standard deviation) over the last 3 days and 7 days prior to answered PROMs. These were denoted with a suffix ‘_m’ or ‘_s’ followed by the numbers ‘3’ or ‘7’, e.g. ‘M10_s3’ would refer to the standard deviation of ‘M10’ actigraphy measure over 3 days prior to the answered PROMs entry. Those were used with the linear fixed-effects models described later.

#### Visualizing actigraphy data

We provided a range of graphical illustrations to visually inspect information that was extracted from actigraphy data. Specifically: (a) *Data summary*, presenting the raw three-dimensional acceleration data, acceleration summary measure (here ROCAM), wrist temperature, and ambient light to have an overview of the actigraphy data per participant; (b) *Actogram*, which concatenates two successive days to present a continuum of 48 consecutive hours with succeeding days stacked vertically, that is particularly useful to visualize sleep regularity; (c) *Colored actogram*^24^, to provide a nuanced continuous overview of acceleration on successive 24-h days stacked vertically, to visualize burst of activity and activity regularity; (d) *PA categories overview*, using stacked histograms per day; (e) *Week-by-week PA chart*, with the time a participant spent on each of the PA categories to evaluate how well people adhere to WHO guidelines regarding weekly PA engagement (https://www.who.int/initiatives/behealthy/physical-activity); (f) *Sleep duration overview*, depicting sleep duration for consecutive days to enable understanding better the sleep duration trajectories, including duration of awakenings and WASO; (g) *Sleep efficiency*, which is defined as the ratio of time asleep over total time in bed per night, providing an estimate of sleep quality which is considered good if it is over 90%, poor if it is between 75 and 85%, whilst below 75% could indicate insomnia symptoms^38^; and (h) *Sleep chart*, which looks like a rose plot conveniently presenting in polar coordinates format (24-h clock) the probabilities that a participant slept and woke up within certain time windows (we chose 20-min windows), to gain an overview of the variability in sleep onset and wake-up times.

Similar visualizations have been previously used but are scattered across the actigraphy and circadian biology research literature. We do not claim novelty in these presentations, however we believe it is the first time we collated these together and have also made some visual adaptations to facilitate interpretation of findings. For example, we included transparent background colors to indicate expected outputs such as in sleep duration, sleep onset and offset, and duration of weekly PA.

### Sector comparisons

We visually explored potential differences between the four sectors (i.e. the four groups of participants). Given the limited sample size we cannot perform detailed statistical comparisons; instead we presented violin plots to gain a tentative indication which might point to potential differences across work sectors (i.e. like a stratification approach to generate hypotheses).

### Statistical analysis

This section summarizes the methods for the statistical analysis and modeling used in the study.

### Statistical associations for the baseline PROMs

We explored the statistical relationship of the raw single-point entries (baseline PROMs) and the same date entries (from the weekly and monthly PROMs) using Spearman correlation coefficients. The correlation coefficient spans the range [− 1… 1], with values close to 0 indicating no correlation, negatives values indicating inversely proportional correlation, and positive values denoting proportional correlation. The higher the magnitude of the coefficient, the stronger the correlation. We used the empirical threshold of |0.3| for the correlation coefficient magnitude to identify relationships which were deemed *statistically strong* in accordance to recommendations for medical and psychological studies^73,74^. To quantify the uncertainty (or confidence) around the computed correlation coefficients we need a strategy given the limited number of available samples (since we only processed baseline PROMs, i.e. a single entry per participant). We used 100 iterations where in each iteration we randomly sampled 90% of the participants, i.e. the equivalent of dropping 10% of participants in each iteration, and computing the correlation coefficient on this subset. Then we computed the standard deviation of the correlation coefficients and the 5th and 95th percentiles across the 100 iterations, which provides the confidence interval for each pairwise statistical relationship. We also assessed whether findings were statistically significant at the *p* = 0.05 level. We computed these correlations both *within* (pairs of items of) the same questionnaire and also *between* (pairs of items of) the different questionnaires. The within questionnaires correlations approach serves to quantify (in a statistical sense) how much internal overlap there is between questionnaire items and in particular quantify the extent questionnaire items drive that particular questionnaire’s total score (e.g. a PSQI item with the global PSQI or a WEMWBS item with the total WEMWBS). The between questionnaires correlations approach serves to quantify how information extracted from a questionnaire item is statistically associated with information in a different area captured by the two questionnaires (e.g. using questionnaire items from PSQI to assess their association strength with wellbeing aspects in WEMWBS).

### Time series associations for the weekly and monthly PROMs

The weekly and monthly questionnaires along with the extracted actigraphy measures from each participant are *multivariate time series data* (e.g. each item scored on different times comprises a *time series*). Therefore, standard approaches used to associate variables such as correlation coefficients are not appropriate for this task, and we need to explore algorithmic approaches which intrinsically account for the fact that e.g. PROMs collected by a participant on consecutive weeks or months are not independent. For this reason, we need to use approaches which often come under the umbrella name *time series analysis* methods. To assess the temporal similarity of time series of the weekly and monthly PROMs, also along with the computed actigraphy measures, we used *cross-correlation*. Cross-correlation has the convenient property that it can provide a measure of similarity between two time series, including identifying whether one leads or lags the other. For a gentle background introduction of cross-correlation we refer to^75^; for a more detailed elaboration please see^76^. We used prior normalization of the time series in all cases to ensure they are comparable, in accordance to standard pre-processing recommendations^76^ before computing the cross-correlation coefficients (XCFs). We aligned time series where required, e.g. to match weekly and monthly questionnaires or match the actigraphy measures (over the 30–40 days collected data each time) and the questionnaires. This was driven by the type of questionnaire: for example, when comparing the actigraphy data with weekly questionnaires we truncated the weekly BJSQ series to match the period of the actigraphy data. This required taking into account of the timeframe used for each questionnaire, e.g. when matching the actigraphy data (about 35 days) with PSQI we used the PSQI entry that was self-reported closest to the last day of actigraphy data collected (given that participants self-report PSQI referring to their sleep over the last month). A similar reasoning was for the weekly questionnaires, matching the PROMs entries closest to every 7th day in the actigraphy data collected (given that weekly PROMs solicit participants’ response over the last week). This process enabled the matching between actigraphy and PROMs to align the time-series for each time a participant wore the wrist-worn wearable, which led to the computation of XCFs.

### Linear mixed-effects models

Linear mixed-effects models are extensions of standard linear regression models. They combine fixed effects (overall trends) with random effects (group variation) to analyze clustered data (in this case, time series from participants). They have the desirable property of accounting for potential autocorrelation within a participant’s time series (e.g. PROMs on adjacent weeks may be more strongly correlated than PROMs further spaced apart). For further background we refer readers to standard authoritative textbooks^77,78^. Linear mixed-effects models have been recently used in similar applications with longitudinal PROMs and actigraphy, e.g. see Edgley et al.^32^. Here, we wanted to explore the statistical modeling relationships to estimate total WEMWBS and global PSQI scores using (i) other PROMs (focusing on work-related items to illustrate the relationships between work and health-related outcomes), (ii) actigraphy-extracted features, and (iii) a combination of PROMs and actigraphy-extracted features. We included basic demographics (age and gender) along with work sector to see whether these have a (conditional) effect on the outcomes. To match actigraphy measures (inferred daily) to PROMs we used a simple strategy computing the average and standard deviation also in the last 3 days and last 7 days prior to when the corresponding PSQI and WEMWBS were registered. These additional features retained the original feature name complemented by a suffix including a letter and a number: we used ‘m’ for average and ‘v’ for standard deviation along with the number ‘3’ or ‘7’ to indicate the prior days.

Specifically, we modeled PSQI and WEMWBS using the following generic equation:

9 $$\:{Y}_{t,j}={\beta\:}_{0}+{\beta\:}_{1}\bullet\:{Day}_{t,j}+\sum\:_{k=1}^{m}{\beta\:}_{k+1}\bullet\:{X}_{k,t,j}+{a}_{1}\bullet\:{Age}_{j}+{a}_{2}\bullet\:{Gender}_{j}+{a}_{3}\bullet\:{WorkSector}_{j}+{u}_{j}+{\epsilon\:}_{t,j}$$

where $\:{Y}_{t,j}$ is the global PSQI or total WEMWBS at time *t* for participant *j*, $\:{a}_{1}$, $\:{a}_{2}$ and $\:{a}_{3}$ are the fixed effects for time-invariant variables (Age, Gender, ‘work sector’). The model coefficients $\:{\boldsymbol{\upbeta\:}=[\beta\:}_{0},\:{\beta\:}_{1},\dots\:]$ represent the fixed effects with $\:{\beta\:}_{0}$ being the global intercept, $\:{\beta\:}_{1}\bullet\:{Day}_{t,j}$ the fixed effect of time (day since study enrolment for participant *j* to capture the overall trend of the study), and the remaining beta coefficients for the corresponding *m* variables used in the model (PROMs and/or actigraphy measures) where $\:{X}_{k,t,j}$ refers to the *k*th variable at time *t* for participant *j*. For the remaining terms, $\:{u}_{j}$ denotes the random effect for participant *j*, and $\:{\epsilon\:}_{t,j}$ is the residual error at time *t* for participant *j*.

We selected a small subset of variables to present to the linear mixed-effect models to mitigate collinearity and illustrate associations. This was guided by a robust, principled feature selection algorithm called Relevance, Redundancy, and Complementarity Trade-off (RRCT), which has been previously demonstrated to be particularly competitive in small and fat datasets^79^. The final model selection was decided through experimentation using the Akaike Information Criterion (AIC)^80^, with lower AIC values denoting a better model. AIC is a relative metric, which depends on the sample size and scaling of the data, so it was only used to compare models on the same dataset (there is no interpretation of the actual AIC value).

All continuous variables were standardized (z-scored) prior to model fitting for direct comparison of effect sizes, whereas the responses were maintained in their original units to preserve interpretability. Since the variables were standardized, this facilitates interpretation: for example, a variable with $\:{\beta\:}_{k}$= 0.5 has twice the predictive power of one with $\:{\beta\:}_{k}$= 0.25. Where appropriate, variables were set to be categorical (gender, participant id, and ‘work sector’). Specifically, these categorical variables were represented in the linear mixed-effects models using dummy variables with effects coding, a scheme which creates one less dummy variable than the number of categories (a standard approach to overcome challenges with the resulting matrix not being full rank when having an intercept and a dummy variable for each possible category). The corresponding coefficient of the remaining ‘hidden’ category is inferred so that the all the coefficients representing the categorical variable amount to zero sum.

In longitudinal studies the intercept often dominates compared to the individual variables inserted into a linear mixed-effects model. To quantify exactly how much the intercept dominates in the model for the prediction of the response, we calculated the Intra-Class Correlation (ICC) which is in the range 0…1 and indicates what percentage of the variance is due to the person (the intercept) compared to the behavior (the variables in the model). High ICC scores (e.g. >0.7) suggest a very stable response, whereas low ICC scores suggest that the variables in the model explain the underlying changes in the response.

### Assessing the internal consistency of the 6-point Likert scale BJSQ

As mentioned above, we modified the standard BJSQ from a 4-point Likert scale to 6-point Likert scale in order to increase its sensitivity. However, this could potentially affect its psychometric properties. For that reason, we need to validate its internal consistency due to the proposed modification. We used the standard approach (Cronbach’s alpha^81^ to assess the internal consistency of the 6-point BJSQ. Cronbach’s alpha coefficient measures the internal consistency, or reliability, of a set of survey items: it quantifies the level of agreement on a standardized 0 to 1 scale. Higher values indicate higher agreement between items, where typically the threshold of 0.8 and above is used to indicate construct reliability. Specifically, we used the weekly BJSQ responses to compute Cronbach’s alpha.

## Supplementary Information

Below is the link to the electronic supplementary material.

Supplementary Material 1: Questionnaires_in_SHAW.xlsx

Supplementary Material 2: Actigraphy_measures.xlsx

Supplementary Material 3: SHAW_study_Supplementary_material.pdf

## Data Availability

The datasets generated and/or analysed during the current study are not publicly available due to confidentiality concerns and because we do not have permission from participants to publicly share, but are available from the corresponding author on reasonable request.

## References

[1] Grundy, E. M. & Murphy, M. Population ageing in Europe. In *Oxford Textbook of Geriatric Medicine* (eds Michel, J. P., Beattie, B. L., Martin, F. C. & Walston, J.) 11–17. 10.1093/med/9780198701590.001.0001 (Oxford University Press, 2017).

[2] Case, A. & Deaton, A. Mortality and morbidity in the 21 st century HHS Public Access. *Brookings Pap Econ. Act.* **2017**, 397–476 (2017). 29033460 10.1353/eca.2017.0005 PMC5640267

[3] McPhee, J. S. et al. Physical activity in older age: perspectives for healthy ageing and frailty. *Biogerontology* **17**, 567–580 (2016). 26936444 10.1007/s10522-016-9641-0 PMC4889622

[4] Santos, A. C., Willumsen, J., Meheus, F., Ilbawi, A. & Bull, F. C. The cost of inaction on physical inactivity to public health-care systems: a population-attributable fraction analysis. *Lancet Glob Heal*. **11**, e32–e39 (2023). 10.1016/S2214-109X(22)00464-8 PMC9748301 36480931

[5] Cunningham, C., O’ Sullivan, R., Caserotti, P. & Tully, M. A. Consequences of physical inactivity in older adults: A systematic review of reviews and meta-analyses. *Scand. J. Med. Sci. Sport*. **30**, 816–827 (2020). 10.1111/sms.13616 32020713

[6] Vanajan, A., Bültmann, U. & Henkens, K. Why do older workers with chronic health conditions prefer to retire early? *Age Ageing*. **49**, 403–410 (2020). 32037457 10.1093/ageing/afz180 PMC7187868

[7] Wilson, D. M. et al. Identifying contemporary early retirement factors and strategies to encourage and enable longer working lives : A scoping review. *Int. J. Older People Nurs.* **15**, e12313 (2020). 32166897 10.1111/opn.12313

[8] Khalid, A. & Syed, J. Mental health and well-being at work: a systematic review of literature and directions for future research. *Hum. Resour. Manag Rev.* **34**, 100998 (2024).

[9] Sonnentag, S., Tay, L. & Nesher Shoshan, H. A review on health and well-being at work: More than stressors and strains. *Pers. Psychol.* **76**, 473–510 (2023).

[10] Michie, S., van Stralen, M. M. & West, R. The behaviour change wheel: a new method for characterising and designing behaviour change interventions. *Implement. Sci.* **6**, 42 (2011). 21513547 10.1186/1748-5908-6-42 PMC3096582

[11] Woodward, K. et al. Beyond mobile apps: A survey of technologies for mental well-being. *IEEE Trans. Affect. Comput.* **13**, 1216–1235 (2022).

[12] Ishaque, S., Karnon, J., Chen, G., Nair, R. & Salter, A. B. A systematic review of randomised controlled trials evaluating the use of patient-reported outcome measures (PROMs). *Qual. Life Res.* **28**, 567–592 (2019). 30284183 10.1007/s11136-018-2016-z

[13] Tsanas, A. et al. Daily longitudinal self-monitoring of mood variability in bipolar disorder and borderline personality disorder. *J. Affect. Disord*. **205**, 225–233 (2016). 27449555 10.1016/j.jad.2016.06.065 PMC5296237

[14] Proudfoot, J. et al. Community attitudes to the appropriation of mobile phones for monitoring and managing depression, anxiety, and stress. *J. Med. Internet Res.* **12**, e64 (2010). 21169174 10.2196/jmir.1475 PMC3057321

[15] Tsanas, A. et al. Clinical insight into latent variables of psychiatric questionnaires for mood symptom self-assessment. *JMIR Ment. Health*. **4**, e15 (2017). 28546141 10.2196/mental.6917 PMC5465382

[16] Palmius, N. et al. London, UK,. A multi-sensor monitoring system for objective mental health management in resource constrained environments. In *Appropriate Healthcare Technologies for Low Resource Settings (AHT 2014)*. 10.1049/cp.2014.0764 (2014).

[17] Edgley, K., Horne, A. W., Saunders, P. T. K. & Tsanas, A. Symptom tracking in endometriosis using digital technologies: Knowns, unknowns, and future prospects. *Cell. Rep. Med.* **4**, 101192 (2023). 37729869 10.1016/j.xcrm.2023.101192 PMC10518625

[18] Knight, S. R. et al. Mobile devices and wearable technology for measuring patient outcomes after surgery: a systematic review. *NPJ Digit. Med.* **4**, 157 (2021). 34773071 10.1038/s41746-021-00525-1 PMC8590052

[19] Puccinelli, P. J. et al. Reduced level of physical activity during COVID-19 pandemic is associated with depression and anxiety levels: an internet-based survey. *BMC Public. Health*. **21**, 425 (2021). 33648487 10.1186/s12889-021-10470-z PMC7919983

[20] Altena, E. et al. Dealing with sleep problems during home confinement due to the COVID-19 outbreak: Practical recommendations from a task force of the European CBT-I Academy. *J. Sleep. Res.* **29**, 1–7 (2020). 10.1111/jsr.13052 32246787

[21] Mace, R. A., Mattos, M. K. & Vranceanu, A. M. Older adults can use technology: why healthcare professionals must overcome ageism in digital health. *Transl. Behav. Med.* **12**, 1102–1105 (2022). 36073770 10.1093/tbm/ibac070 PMC9494377

[22] Chen, C., Ding, S. & Wang, J. Digital health for aging populations. *Nat. Med.* **29**, (2023). 10.1038/s41591-023-02391-8 37464029

[23] Piwek, L., Ellis, D. A., Andrews, S. & Joinson, A. The rise of consumer health wearables: Promises and barriers. *PLoS Med.* **13**, e1001953 (2016). 26836780 10.1371/journal.pmed.1001953 PMC4737495

[24] Tsanas, A., Woodward, E. & Ehlers, A. Objective characterization of activity, sleep, and circadian rhythm patterns using a wrist-worn actigraphy sensor: insights into post-traumatic stress disorder. *JMIR mHealth uHealth*. **8**, e14306 (2020). 32310142 10.2196/14306 PMC7199134

[25] Tsanas, A. Accurately inferring physical activity levels and sleep from wrist-worn actigraphy recordings with sample rates as low as 10 Hz. *IEEE Access.* **13**, 27257–27267 (2025).

[26] Doherty, A. et al. Large scale population assessment of physical activity using wrist worn accelerometers: the UK Biobank study. *PLoS One*. **12**, e0169649 (2017). 28146576 10.1371/journal.pone.0169649 PMC5287488

[27] Walmsley, R. et al. Reallocation of time between device-measured movement behaviours and risk of incident cardiovascular disease. *Br. J. Sports Med.*10.1136/bjsports-2021-104050 (2021). 10.1136/bjsports-2021-104050 PMC9484395 34489241

[28] Saint-Maurice, P. F. et al. Associations between actigraphy-measured sleep duration, continuity, and timing with mortality in the UK Biobank. *Sleep* **47**, 1–12 (2024). 10.1093/sleep/zsad312 PMC10925955 38066693

[29] Patten, T. et al. The all of us research program’s wearables dataset. *Nat. Med.*10.1038/s41591-026-04352-3 (2026). 42045581 10.1038/s41591-026-04352-3 PMC13278962

[30] Edgley, K., Chun, H. Y. Y., Whiteley, W. N. & Tsanas, A. Temperature and sleep data using wrist-worn wearables. *Sensors* **23**, 1069 (2023). 36772109 10.3390/s23031069 PMC9920931

[31] Chun, H. Y. Y. et al. Telemedicine cognitive behavioral therapy for anxiety after stroke: proof-of-concept randomized controlled trial. *Stroke* **51**, 2297–2306 (2020). 32576090 10.1161/STROKEAHA.120.029042 PMC7382539

[32] Edgley, K., Saunders, P. T. K., Whitaker, L. H. R., Horne, A. W. & Tsanas, A. Insights into endometriosis symptom trajectories and assessment of surgical intervention outcomes using longitudinal actigraphy. *NPJ Digit. Med.* **8**, e236 (2025). 10.1038/s41746-025-01629-8 PMC12048534 40316659

[33] Ancoli-Israel, S. et al. The role of actigraphy in the study of sleep and circadian rhythms. *Sleep* **26**, 342–392 (2003). 12749557 10.1093/sleep/26.3.342

[34] Tsanas, A. Investigating wrist-based acceleration summary measures across different sample rates towards 24-hour physical activity and sleep profile assessment. *Sensors* **22**, 6152 (2022). 36015910 10.3390/s22166152 PMC9413015

[35] Vitiello, M. V., Larsen, L. H. & Moe, K. E. Age-related sleep change: Gender and estrogen effects on the subjective-objective sleep quality relationships of healthy, noncomplaining older men and women. *J. Psychosom. Res.* **56**, 503–510 (2004). 15172206 10.1016/S0022-3999(04)00023-6

[36] Lok, R., Qian, J. & Chellappa, S. L. Sex differences in sleep, circadian rhythms, and metabolism: implications for precision medicine. *Sleep. Med. Rev.* **75**, 101926 (2024). 38564856 10.1016/j.smrv.2024.101926

[37] Bixler, E. O. et al. Women sleep objectively better than men and the sleep of young women is more resilient to external stressors: Effects of age and menopause. *J. Sleep. Res.* **18**, 221–228 (2009). 19302341 10.1111/j.1365-2869.2008.00713.x PMC3594776

[38] Kryger, M. H., Roth, T. & Dement, W. C. *Principles and Practice of Sleep Medicine* (Saunders, 2021).

[39] Maas, C. J. M. & Hox, J. J. Sufficient sample sizes for multilevel modeling. *Methodology* **1**, 86–92 (2005).

[40] Hox, J. J., Moerbeek, M. & van de Schoot, R. *Multilevel Analysis.Pdf* (Routledge, 2018).

[41] Diggle, P. J., Heagerty, P. J., Liang, K. Y. & Zeger, S. L. *Analysis of Longitudinal Data* (Oxford University Press, 2002).

[42] Churruca, K. et al. Patient-reported outcome measures (PROMs): a review of generic and condition-specific measures and a discussion of trends and issues. *Heal Expect.* **24**, 1015–1024 (2021). 33949755 10.1111/hex.13254 PMC8369118

[43] Pratap, A. et al. Indicators of retention in remote digital health studies: a cross-study evaluation of 100,000 participants. *NPJ Digit. Med.* **3**, 21 (2019). 10.1038/s41746-020-0224-8 PMC7026051 32128451

[44] Lehmann, J. et al. Adherence to patient-reported symptom monitoring and subsequent clinical interventions for patients with multiple myeloma in outpatient care: Longitudinal observational study. *J. Med. Internet Res.* **25**, e46017 (2023). 37606979 10.2196/46017 PMC10481208

[45] Meyers, A., Buqammaz, M. & Yang, H. Cross-recurrence analysis for pattern matching of multidimensional physiological signals. *Chaos* **30**, 123125 (2020). 33380053 10.1063/5.0030838

[46] Fernández-Montes, A. et al. SimilarityTS: Toolkit for the evaluation of similarity for multivariate time series. *SoftwareX* **24**, 101527 (2023).

[47] Dove, S., Böhm, M., Freeman, R., Jellesmark, S. & Murrell, D. J. A user-friendly guide to using distance measures to compare time series in ecology. *Ecol. Evol.* **13**, e10520 (2023). 37809360 10.1002/ece3.10520 PMC10551742

[48] Mueller, M. Dynamic Time Warping. in Information Retrieval for Music and Motion 69–84 (Springer, Berlin, doi:10.1007/978-3-540-74048-3_4. (2007).

[49] Tsanas, A. *Accurate Telemonitoring of Parkinson’s Disease Using Nonlinear Speech Signal Processing and Statistical Machine Learning* (University of Oxford, 2012).

[50] Neal, Margaret, B., Chapman, N. J., Ingersoll-Dayton, B. & Emlen, A. C. *Balancing Work and Caregiving for Children, Adults, and Elders* (SAGE, 1993).

[51] Lloyd, L. et al. Look after yourself : active ageing, individual responsibility and the decline of social work with older people in the UK. *Eur. J. Soc. Work*. **17**, 322–335 (2014).

[52] Ng, E. S. W. & Law, A. Keeping up! Older workers’ adaptation in the workplace after age 55. *Can. J. Aging*. **33**, 1–14 (2014). 24345532 10.1017/S0714980813000639

[53] Hu, S. et al. Digital health: current applications, challenges, and future directions for enhancing healthcare quality and safety. *Front. Public. Heal*. 10.3389/fpubh.2025.1646802 (2025). 41089861 10.3389/fpubh.2025.1646802 PMC12516163

[54] Donnelly, J. M. et al. From reactive to proactive: continuous protein monitoring for preventive health care. *Sci. (80-)*. **389**, eady6497 (2025). 10.1126/science.ady6497 PMC12687573 40997178

[55] Abdulmalek, S. et al. IoT-based healthcare-monitoring system towards improving quality of life: a review. *Healthcare* **10**, 1993 (2022). 36292441 10.3390/healthcare10101993 PMC9601552

[56] Hachenberger, J. et al. Investigating associations between physical activity, stress experience, and affective wellbeing during an examination period using experience sampling and accelerometry. *Sci. Rep.* **13**, 1–10 (2023). 37258597 10.1038/s41598-023-35987-8 PMC10232510

[57] Thurman, S. M. et al. Individual differences in compliance and agreement for sleep logs and wrist actigraphy: A longitudinal study of naturalistic sleep in healthy adults. *PLoS One*. **13**, 1–23 (2018). 10.1371/journal.pone.0191883 PMC5788380 29377925

[58] Schütz, N. et al. A systems approach towards remote health-monitoring in older adults: introducing a zero-interaction digital exhaust. *NPJ Digit. Med.* **5**, 116 (2022). 35974156 10.1038/s41746-022-00657-y PMC9381599

[59] Harris, P. A. et al. The REDCap consortium: Building an international community of software platform partners. *J. Biomed. Inf.* **95**, 103208 (2019). 10.1016/j.jbi.2019.103208 PMC7254481 31078660

[60] Buysse, D. J., Reynolds, C. F., Monk, T. H., Berman, S. R. & Kupfer, D. J. The Pittsburgh sleep quality Index: a new instrument psychiatric practice and research. *Psychiatry Res.* **28**, 193–213 (1989). 2748771 10.1016/0165-1781(89)90047-4

[61] Watanabe, K. et al. Usage of the brief job stress questionnaire: A systematic review of a comprehensive job stress questionnaire in Japan from 2003 to 2021. *Int. J. Environ. Res. Public. Health* **20**, (2023). 10.3390/ijerph20031814 PMC9914747 36767182

[62] Tennant, R. et al. The Warwick-Edinburgh mental well-being scale (WEMWBS): development and UK validation. *Health Qual. Life Outcomes*. **5**, 63 (2007). 18042300 10.1186/1477-7525-5-63 PMC2222612

[63] Ng Fat, L., Scholes, S., Boniface, S., Mindell, J. & Stewart-Brown, S. Evaluating and establishing national norms for mental wellbeing using the short Warwick–Edinburgh Mental Well-being Scale (SWEMWBS): findings from the Health Survey for England. *Qual. Life Res.* **26**, 1129–1144 (2017). 27853963 10.1007/s11136-016-1454-8 PMC5376387

[64] Parker, G. B. & Hyett, M. P. Measurement of well-being in the workplace: The development of the work well-being questionnaire. *J. Nerv. Ment Dis.* **199**, 394–397 (2011). 21629018 10.1097/NMD.0b013e31821cd3b9

[65] Cella, D. et al. The patient-reported outcomes measurement information. *Med. Care*. **45**, 3–11 (2007). 17443116 10.1097/01.mlr.0000258615.42478.55 PMC2829758

[66] van Hees, V. T. et al. Separating movement and gravity components in an acceleration signal and implications for the assessment of human daily physical activity. *PLoS One*. **8**, e61691 (2013). 23626718 10.1371/journal.pone.0061691 PMC3634007

[67] Zhou, S. M. et al. Classification of accelerometer wear and non-wear events in seconds for monitoring free-living physical activity. *BMJ Open.* **5**, e007447 (2015). 25968000 10.1136/bmjopen-2014-007447 PMC4431141

[68] van Hees, V. T. et al. A novel, open access method to assess sleep duration using a wrist-worn accelerometer. *PLoS One*. **10**, e0142533 (2015). 26569414 10.1371/journal.pone.0142533 PMC4646630

[69] van Hees, V. T. *Package ‘ GGIR ’*. (2017).

[70] Blume, C., Santhi, N. & Schabus, M. nparACT’ package for R: A free software tool for the non-parametric analysis of actigraphy data. *MethodsX* **3**, 430–435 (2016). 27294030 10.1016/j.mex.2016.05.006 PMC4890079

[71] Faedda, G. L. et al. Actigraph measures discriminate pediatric bipolar disorder from attention-deficit/hyperactivity disorder and typically developing controls. *J. Child. Psychol. Psychiatry Allied Discip*. **6**, 706–716 (2016). 10.1111/jcpp.12520 PMC4873411 26799153

[72] Kaiser, J. F. On a simple algorithm to calculate the ‘energy’ of a signal. In *ICASSP*, 381–384 (1990).

[73] Tsanas, A., Little, M. A. & McSharry, P. E. A methodology for the analysis of medical data. In *Handbook of Systems and Complexity in Health* (eds Sturmberg, J. P. & Martin, C. M.) 113–125 (Springer, 2013).

[74] Hemphill, J. F. Interpreting the magnitudes of correlation coefficients. *Am. Psychol.* **58**, 78–79 (2003). 12674822 10.1037/0003-066x.58.1.78

[75] Derrick, T. R. & Thomas, J. M. Time series analysis: the cross-correlation function. In *Innovative Analyses of Human Movement* (ed. Stergiou, N.) 189–205 (Human Kinetics, 2004).

[76] Chatfield, C. & Xing, H. *The Analysis of Time Series* (CRC, 2019).

[77] Funatogawa, I. & Funatogawa, T. *Longitudinal Data Analysis Autoregressive Linear Mixed Effects Models* (Springer, 2018).

[78] Gałecki, A. & Burzykowski, T. *Linear Mixed-Effects Models Using R* (Springer, 2013).

[79] Tsanas, A. Relevance, redundancy and complementarity trade-off (RRCT): a principled, generic, robust feature selection tool. *Patterns* **3**, 100471 (2022). 35607618 10.1016/j.patter.2022.100471 PMC9122960

[80] Hastie, T., Tibshirani, R. & Friedman, J. *Elements of Statistical Learning* (Springer, 2009).

[81] Cronbach, L. J. Coefficient alpha and the internal structure of tests. *Psychometrika* **16**, 297–334 (1951).

