# Supplementary material for: Interrelations of work with health and wellbeing on a 50+ year old workforce assessed using longitudinal self-reports and actigraphy
Source: Sci Rep. 2026 Jul 28;16:23215. doi: 10.1038/s41598-026-58229-z (PMC13415521; doi:10.1038/s41598-026-58229-z)
Supplement: Supplementary file 3 — Supplementary Material 3: SHAW_study_Supplementary_material.pdf [file 41598_2026_58229_MOESM3_ESM.pdf]

Supplementary Material

Interrelations of work with health and wellbeing on a 50+ year old workforce assessed using longitudinal self reports and actigraphy

Athanasios Tsanas[1,2*], Belinda Steffan[3], Billy Dixon[4], Kiersten Hay[4], Larissa Pschetz[4], Jakov Jandric[3], Wendy Loretto[3]

[1] Usher Institute, University of Edinburgh, Edinburgh, UK

[2] School of Mathematics, University of Edinburgh, Edinburgh, UK

[3] Business School, University of Edinburgh, Edinburgh, UK

[4] Design Informatics, University of Edinburgh, Edinburgh, UK

*** Correspondence**: (A. Tsanas): Usher Institute, University of Edinburgh, Edinburgh, UK; atsanas@ed.ac.uk

Power analysis

When designing the study we carefully considered the number of participants we would aim to recruit bearing in mind that high-frequency reporting (weekly) over a long duration (one year) is a high-burden task. While traditional power analyses prioritize the number of study participants (*N*), studies of symptom variability require longitudinal observations (*n*) to stabilize within-person variance estimates. In particular, we need to keep in mind that a key objective was to monitor longitudinal trajectories rather than assess group differences. This has important implications for the computation formulas and the longitudinal component needs to be explicitly factored in.

The standard longitudinal power formula to calculate the required number of participants *N* for a study with *n* repeated measures to detect an effect size *d* with a specific power (1-β) and significance level (α) is [1]:

$$N = \frac{2 \cdot \left(z_\alpha + z_\beta\right)^2 \cdot \sigma^2}{n \cdot d^2} \times [1 + (n - 1) \cdot \rho]$$

where z_α and z_β are the z-scores which denote the quantiles of a standard Gaussian distribution and are provided in statistical tables (z_α =1.96 for significance α=0.05, and z_β =0.84 for 80% power), σ^2 is the variance of the scores, ρ is the correlation of measurements within participant, and *d* is the effect size. Substituting conservative values in the formula: $n = 35$, i.e. expecting on average 35 weekly PROMs per participant to account for missing entries in a year (a total of 52), $\rho = 0.5$ which assumes 50% of the variance is between-person "trait" and 50% is within-person "state", and the standardized difference $\Delta = d/\sigma = 0.5$ (to detect a change *d* that is equal to half of one standard deviation σ), we obtain $N \cong 32$.

Using the sample size calculation for longitudinal data we determined that a total of 32 participants would be sufficient to detect a medium effect (*d*=0.5) with 80% power. Therefore, we decided that a target participant size of 55 would comfortably meet this minimum recommended sample size, whilst allowing for potential participants who might drop out of the study. Our resulting sample of *N*=45 provides additional statistical headroom, ensuring robust estimates even if the correlation between PROMs is higher than expected or to account for potential attrition over the one-year study period.

Further Results

This section provides some further findings in addition to the Results presented in the manuscript.

Running adherence

Figure 1 in the main manuscript presented the overall adherence for PROMs and wearables. It is often useful to understand if there are specific patterns in adherence, and one convenient way is to visualize the running adherence, i.e. the percentage of PROMs entered over the expected number of PROMs that should have been entered by that point in the study. This serves as an indication to identify e.g. if participants were very adherent in the beginning of the study and subsequently did not register responses, or if there was a consistent pattern of missing intermittent PROMs etc. Intuitively and on the basis of findings presented in previous studies [2,3], we expected that adherence overall would be dropping with time, at least for some participants. Figure S1 presents the running adherence for the weekly PROMs for all participants in the study. We remark that the vast majority of participants retained an adherence in the range 50-80% until the end of the PROMs data collection, which is promising. As expected, there was some variation in the running adherence, however for most participants this was relatively steady beyond the first few months of participation.

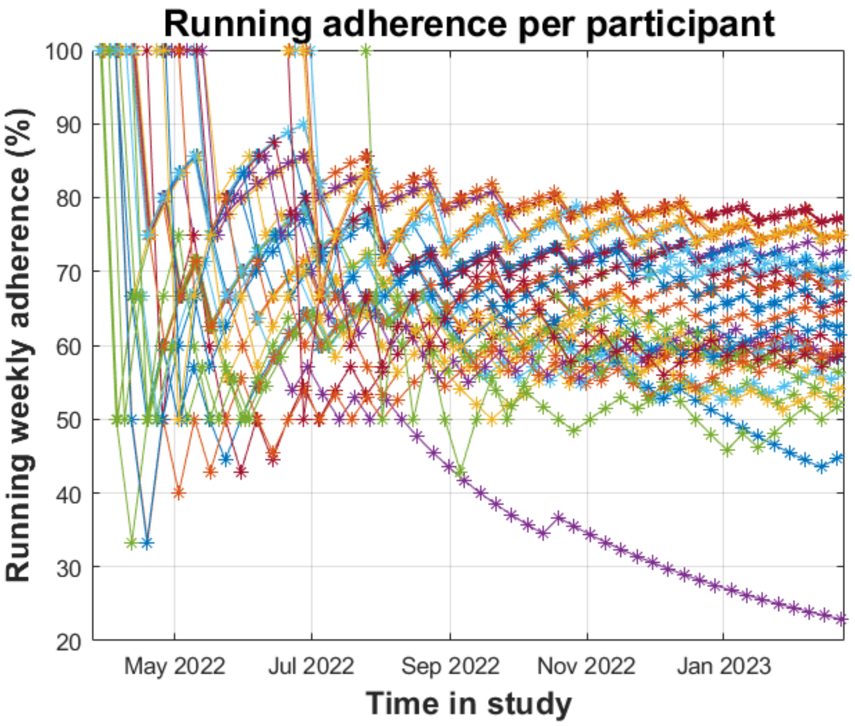

Figure S1: *Running adherence per participant for the weekly PROMs. Each line indicates a unique study participant from their starting point in the study until the time they completed participation.*

Exploring PROMs further at baseline and longitudinally

Figure 2 in the manuscript presented the baseline characteristics across the different questionnaires used in the study. Exploring strategies to stratify the data and discern whether there are patterns for subgroups may often provide useful insights. Given the relatively limited number of participants in SHAW, we are pragmatically restricted by the levels of stratification we can study. Indicatively, Figure S2 presents the gender-stratified data across the questionnaires. Overall, men had slightly worse PSQI. In terms of sleep (as self-reported in PSQI), the main difference is that men slept somewhat less (sleep duration), however women slept considerably less efficiently according to these self-reports. Regarding work aspects (as self-reported in BJSQ), women reported having more work to do and the work environment was not challenging (different departments getting along) compared to men who reported that there were some overall challenges across departments. Self-reporting on wellbeing (WEMWBS), men were more confident that they were thinking clearly but were broadly less close to other people. Women had greater variability in overall wellbeing, although broadly this was fairly similar across men and women. The general feeling from these plots is that there are some nuanced differences in specific items, however the overall scores in the questionnaires are broadly similar across both males and females.

Figure S3 presents the trajectories of the weekly and monthly PROMs for an indicative participant, as an illustration of the underlying nature of the time series data (longitudinal PROMs) in the study. We use this as an exemplar to illustrate how we might build a participant portrait on the basis of the provided PROMs. Using the data from P2 in our study as an example, we can see that in BJSQ they reported they were fairly active and energetic, with no particularly troubling symptoms of their mental health at any point, and occasional problems with sleep. However, in PSQI the participant indicated that their sleep duration and sleep efficiency were substantially reduced almost consistently throughout the study, and that is reflected in the global PSQI being well above the threshold indicating poor sleep (>5). Their wellbeing was fairly moderate with WEMWBS items indicating the participant was probably introvert reporting they are not very close to people or feeling particularly loved. They self-reported they engaged in PA weekly (WWQB-B), with good work-life balance, having the required tools and facilities to do their work and had no problems with being in painful positions (WWQB-I and WWQB-J).

Figure S4 presents the heatmaps for the questionnaires used at baseline, weekly, and monthly to have an overall impression of the statistical associations between pairs of items both within the same questionnaire and across items of other questionnaires. By visual inspection, it is clear there are some strong associations both within questionnaires and also between different questionnaires. Unsurprisingly, the associations were generally stronger within items of the same questionnaire.

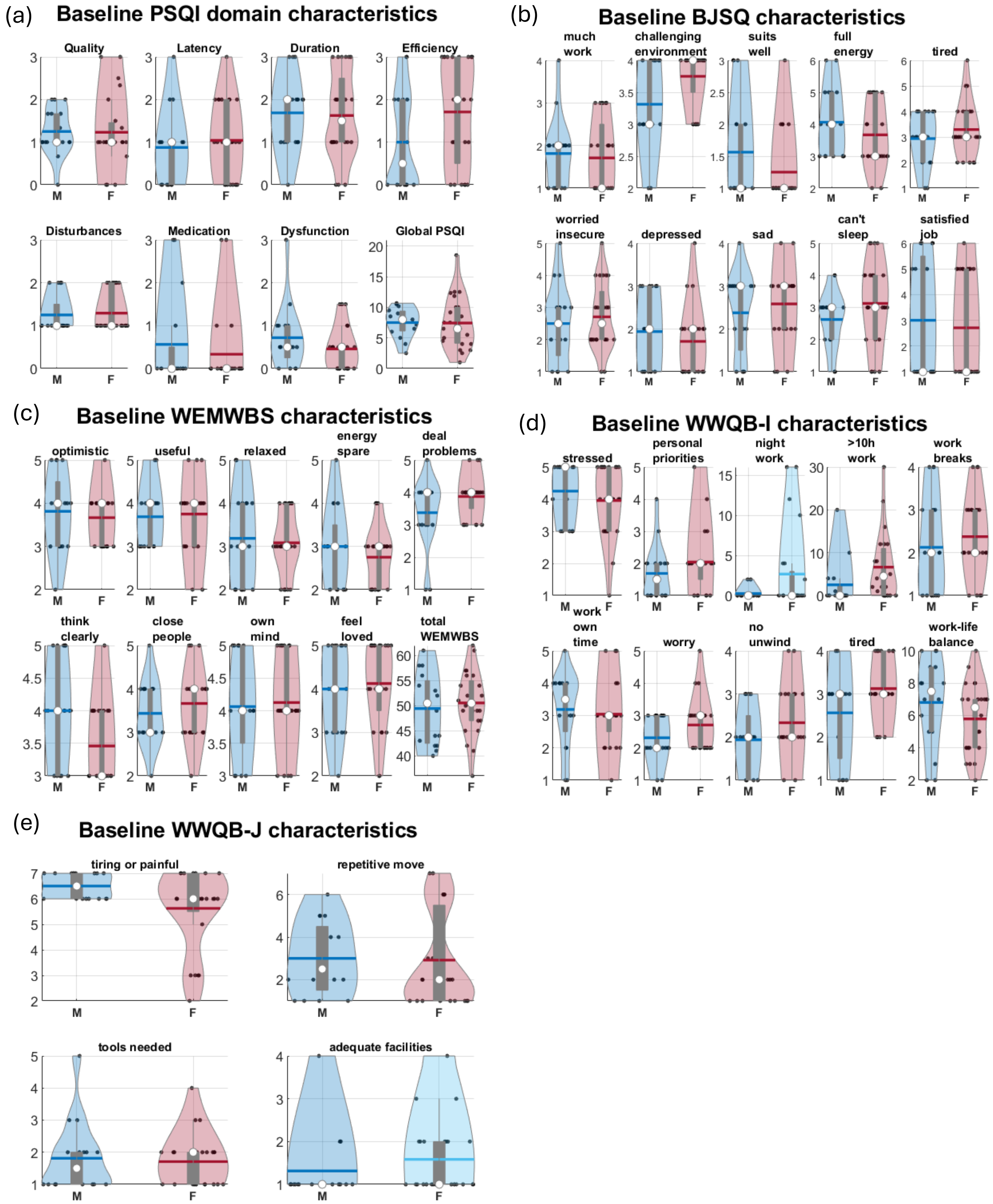

Figure S2: *Violin plots presenting the baseline characteristics following gender stratification, across the different questionnaires: (a) PSQI, (b) BJSQ, (c) WEMWBS, (d) WWQB-I, and (e) WWQB-J. We pooled data from the different work sectors together before stratifying into males (M) and females (F), as denoted in the plots.*

(a)

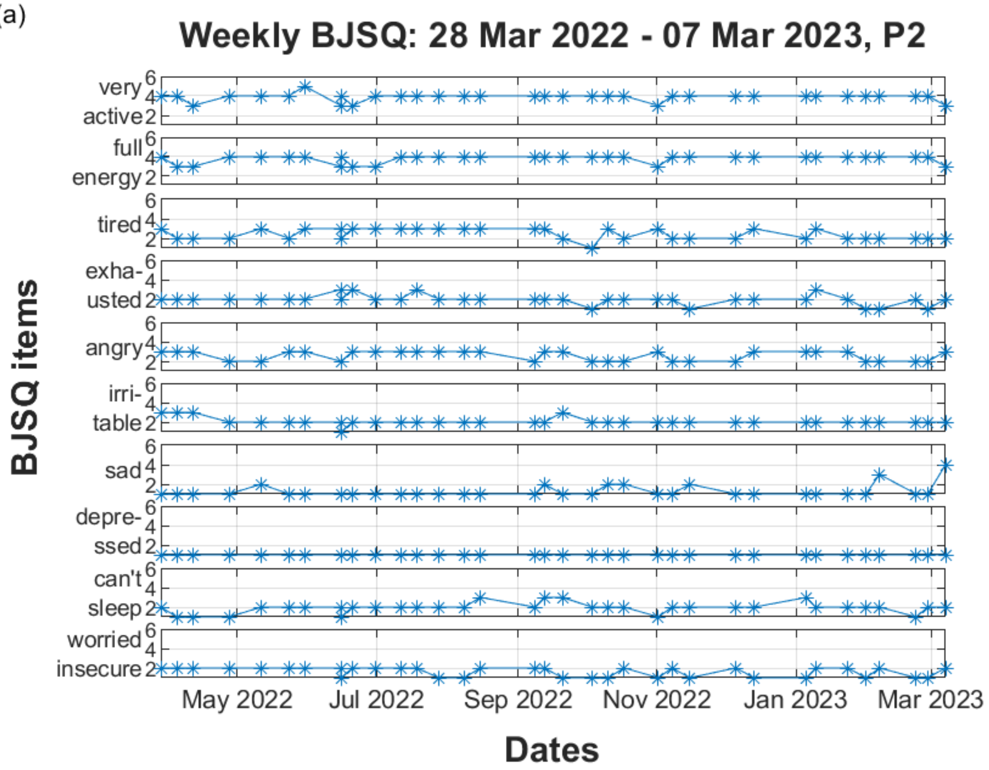
Weekly BJSQ: 28 Mar 2022 - 07 Mar 2023, P2
BJSQ items
very active
full energy
tired
exha-usted
angry
irri-table
sad
depre-ssed
can't sleep
worried insecure
May 2022
Jul 2022
Sep 2022
Nov 2022
Jan 2023
Mar 2023
Dates

(b)

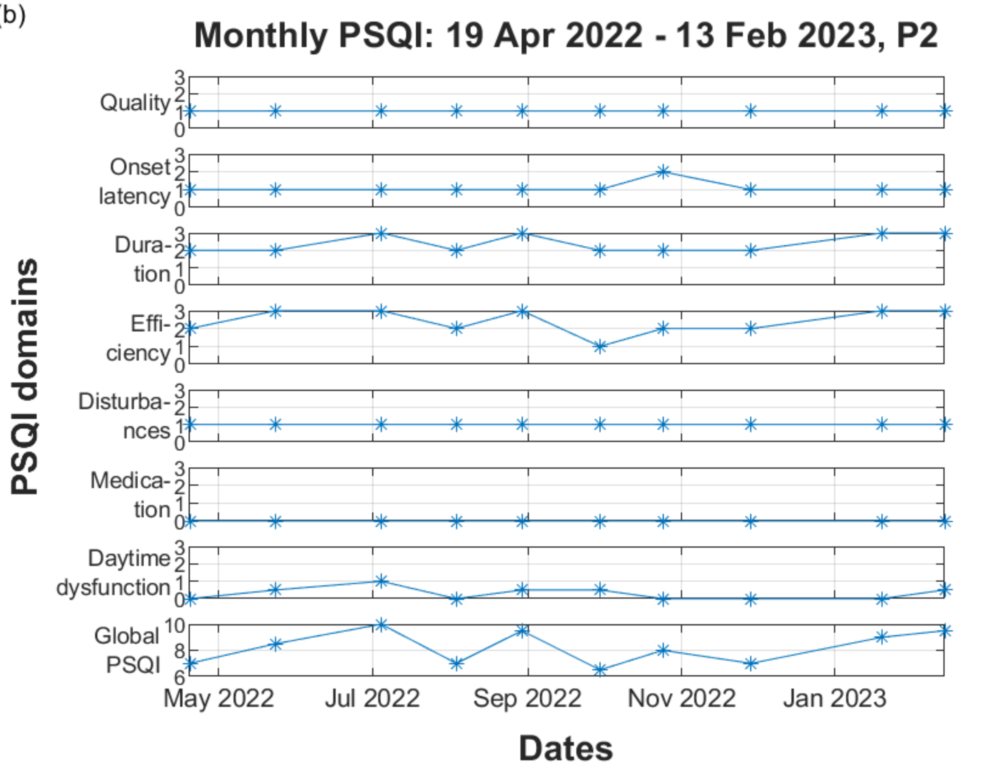
Monthly PSQI: 19 Apr 2022 - 13 Feb 2023, P2
PSQI domains
Quality
Onset latency
Dura-tion
Effi-ciency
Disturba-nces
Medica-tion
Daytime dysfunction
Global PSQI
May 2022
Jul 2022
Sep 2022
Nov 2022
Jan 2023
Dates

(c)

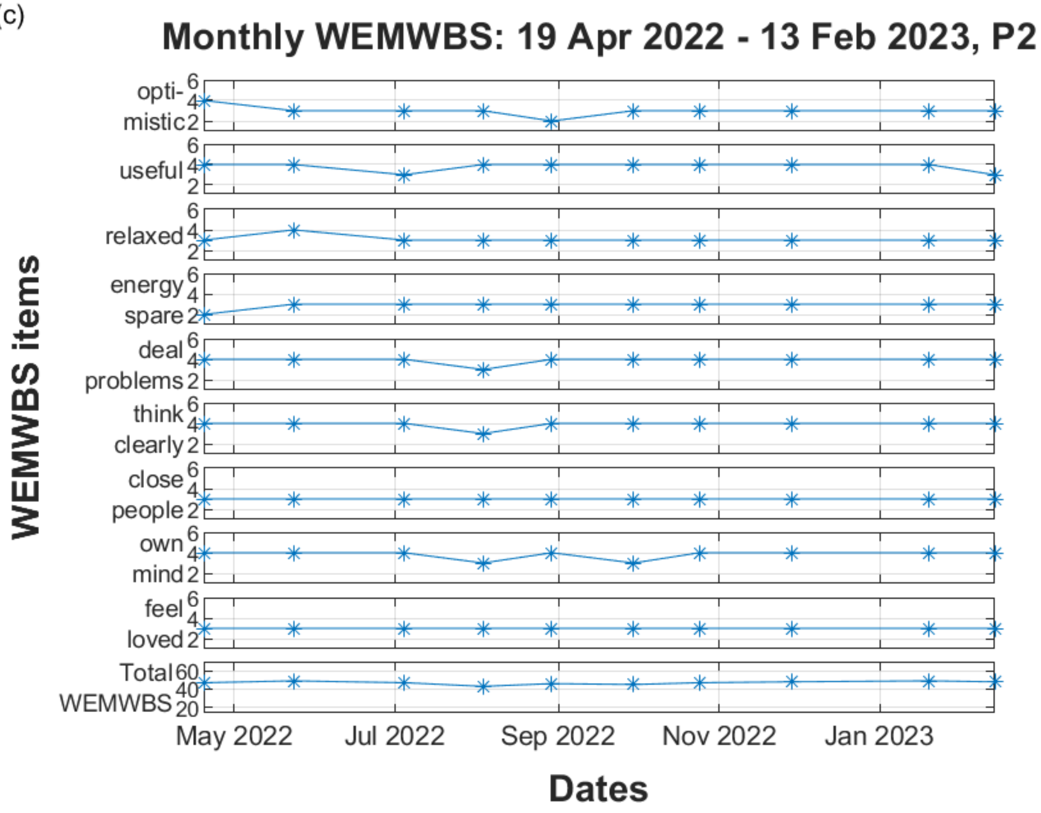

Monthly WEMWBS: 19 Apr 2022 - 13 Feb 2023, P2
WEMWBS items
optimistic
useful
relaxed
energy spare
deal problems
think clearly
close people
own mind
feel loved
Total WEMWBS
May 2022
Jul 2022
Sep 2022
Nov 2022
Jan 2023
Dates

(d)

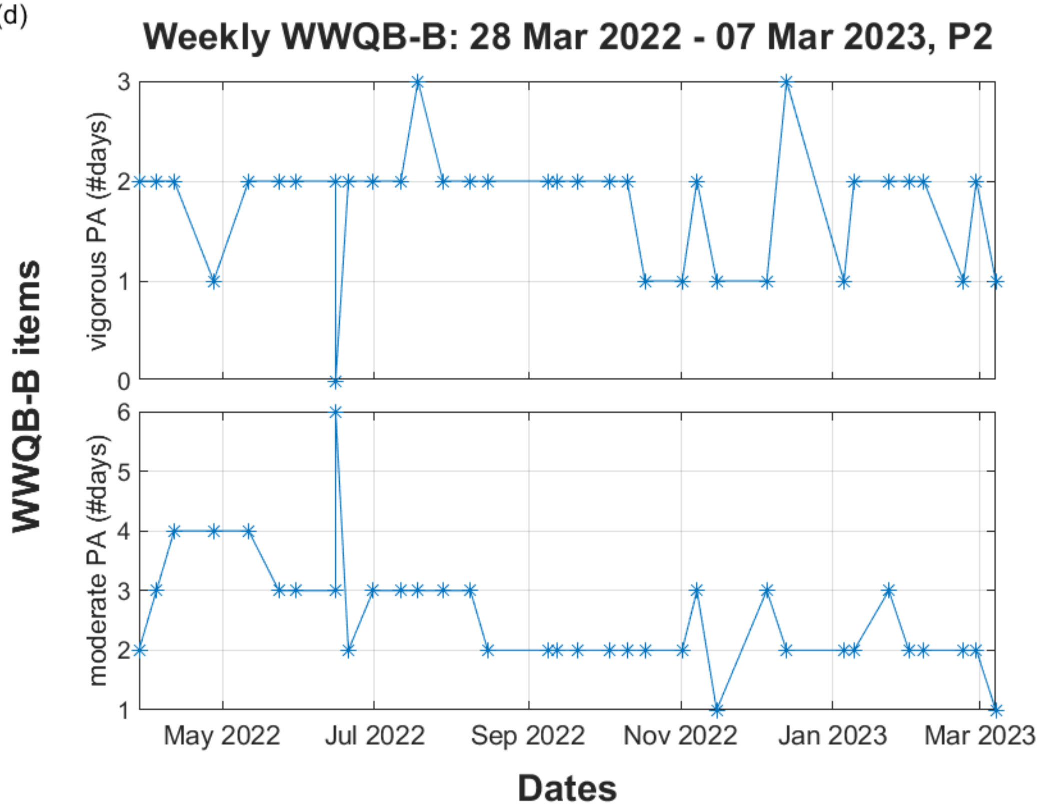

Weekly WWQB-B: 28 Mar 2022 - 07 Mar 2023, P2
WWQB-B items
vigorous PA (#days)
moderate PA (#days)
May 2022
Jul 2022
Sep 2022
Nov 2022
Jan 2023
Mar 2023
Dates

(e)

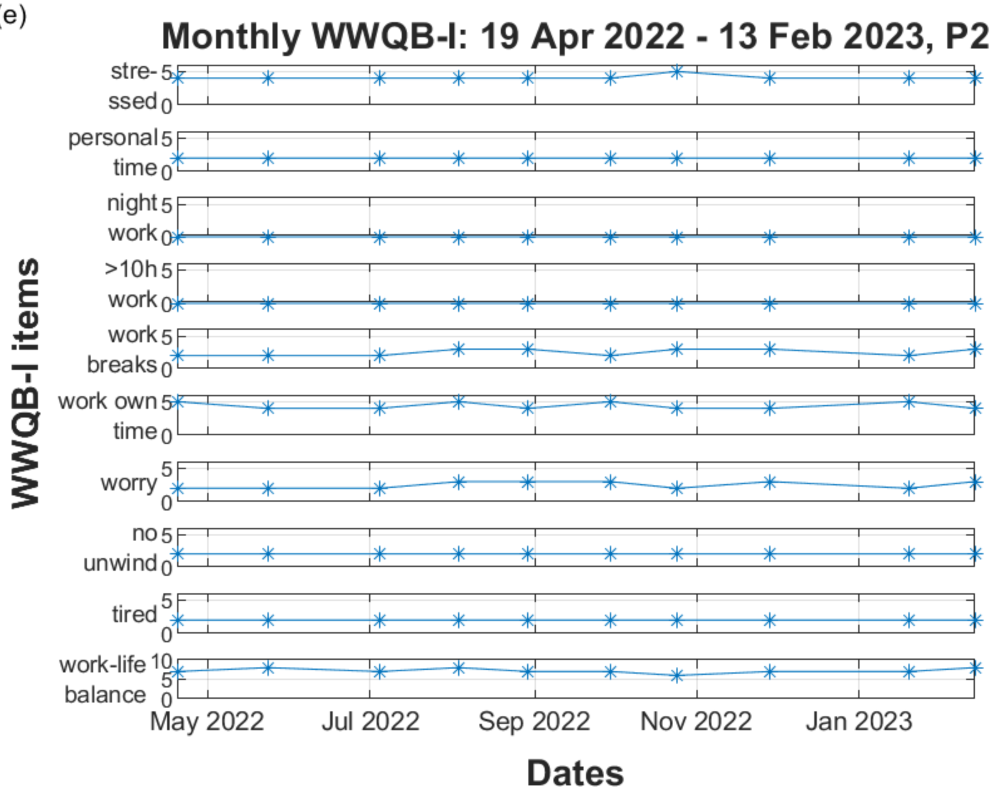

(f)

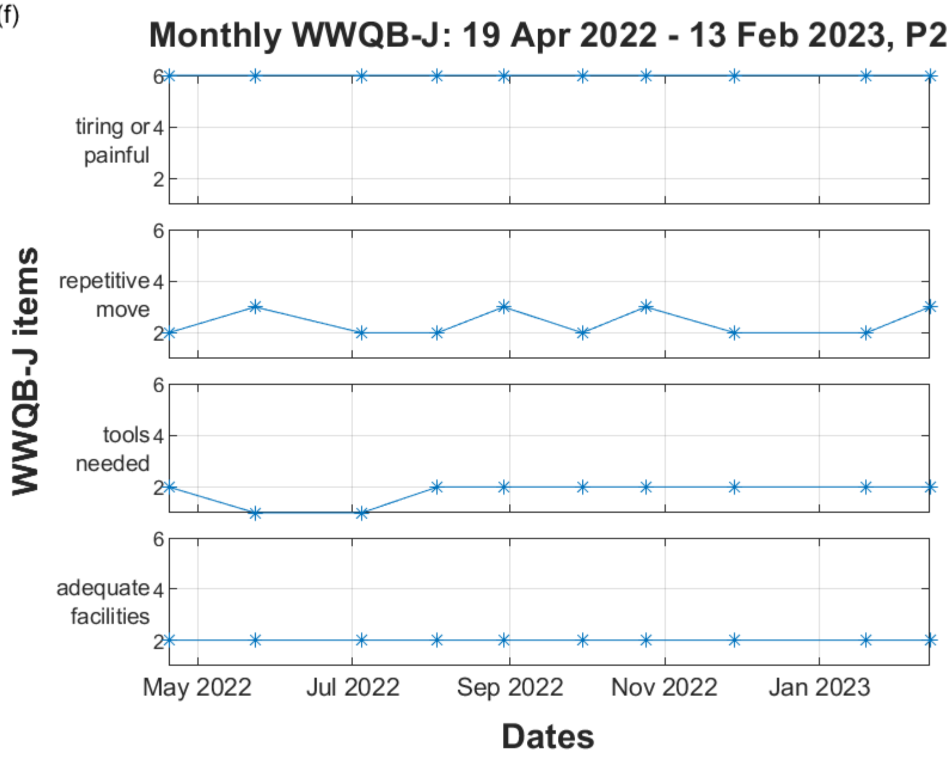

Figure S3: *Trajectories of the weekly and monthly questionnaires used in the study, for an indicative study participant (P2).*

(a)

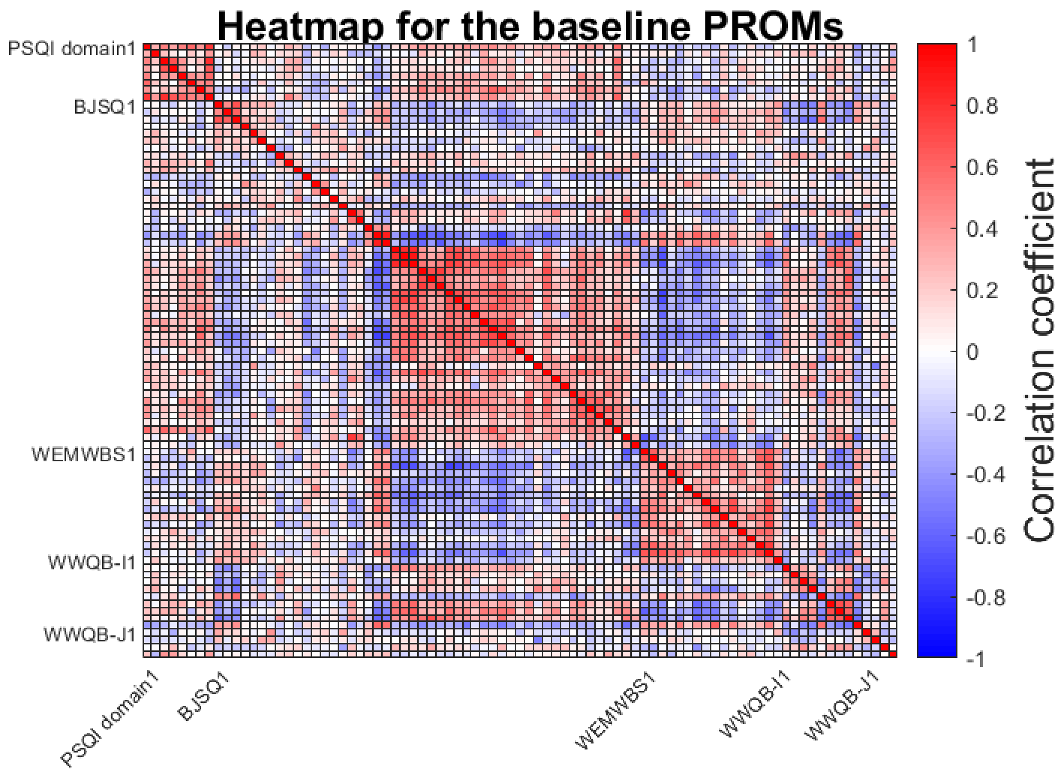
Heatmap for the baseline PROMs
PSQI domain1
BJSQ1
WEMWBS1
WWQB-I1
WWQB-J1
Correlation coefficient
1
0.8
0.6
0.4
0.2
0
-0.2
-0.4
-0.6
-0.8
-1

(b)

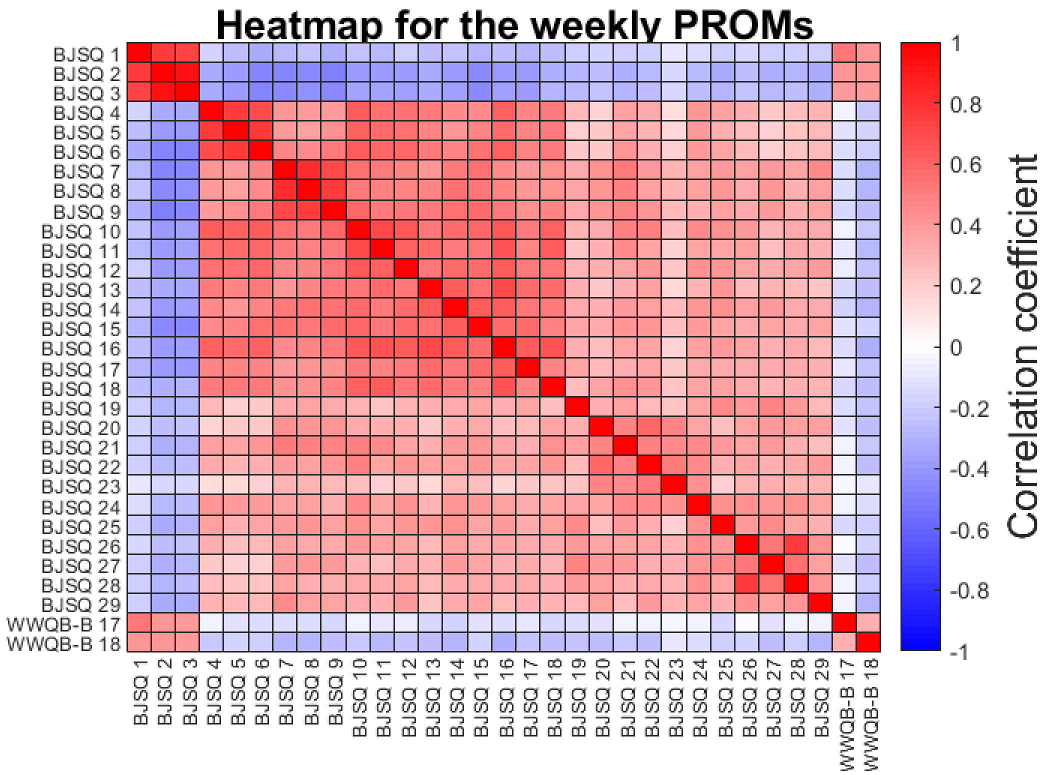
Heatmap for the weekly PROMs
BJSQ 1
BJSQ 2
BJSQ 3
BJSQ 4
BJSQ 5
BJSQ 6
BJSQ 7
BJSQ 8
BJSQ 9
BJSQ 10
BJSQ 11
BJSQ 12
BJSQ 13
BJSQ 14
BJSQ 15
BJSQ 16
BJSQ 17
BJSQ 18
BJSQ 19
BJSQ 20
BJSQ 21
BJSQ 22
BJSQ 23
BJSQ 24
BJSQ 25
BJSQ 26
BJSQ 27
BJSQ 28
BJSQ 29
WWQB-B 17
WWQB-B 18
Correlation coefficient
1
0.8
0.6
0.4
0.2
0
-0.2
-0.4
-0.6
-0.8
-1

(c)

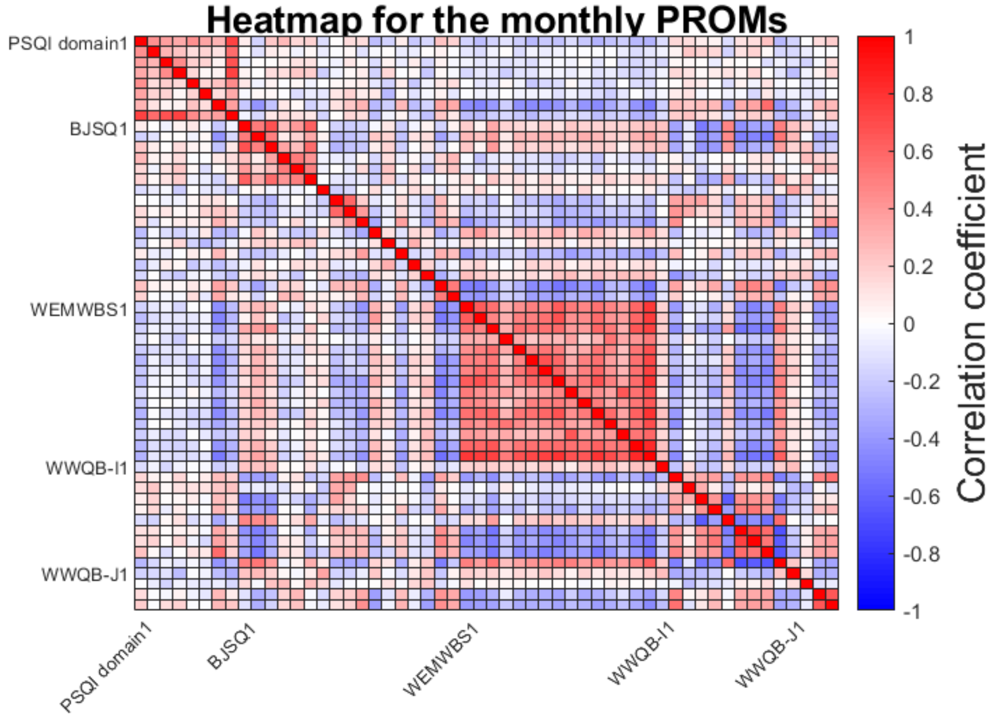

Figure S4: *Heatmaps for the questionnaires used at (a) baseline, (b) weekly, and (c) monthly. To avoid over-cluttering the figures with item labels (baseline and monthly), we only retained the first label for each of the questionnaires used. For PSQI we used the PSQI domains and the global PSQI.*

Figure S5 illustrates joint time series (global PSQI and WEMWBS) for some indicative participants, selected to demonstrate the variability of the underlying trajectory relationships. Following visual inspection, overall wellbeing (as assessed using total WEMWBS) does not have a consistent pattern as a function of sleep problems as self-reported using PSQI for different participants. For some participants we can see that problems in sleep are directly linked with reduced overall wellbeing (e.g. for P1), however for others (e.g. P47) it seems this is not the case.

The individualized association between the global PSQI and total WEMWBS quantified using the cross-correlation coefficient (XCF) at zero lag across all participants is summarized in Figure S6, where for convenience we have color-marked males and females. There is no obvious pattern whether global PSQI and total WEMWBS are proportional or inversely proportional for either males or females: it is clear this is very much participant-specific, underlining the need to be developing personalized models to understand individualized patterns and trajectories that collectively take into account different aspects of daily living (including sleep).

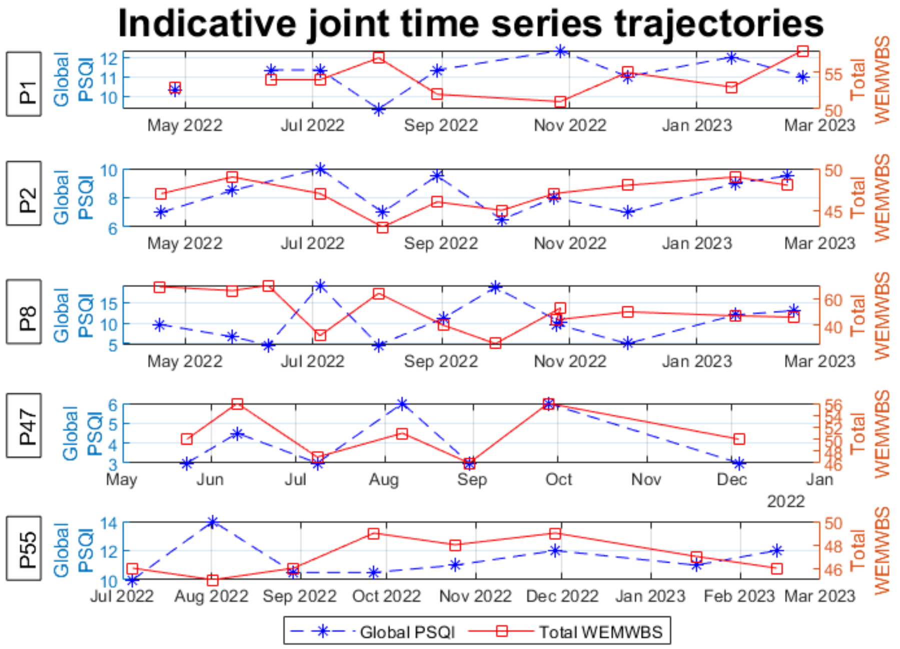

Figure S5: *Indicative joint longitudinal PROMs (global PSQI and total WEMWBS, collected monthly) for five participants to visually inspect trajectory relationships. On the left hand side we indicate the study participant identifier.*

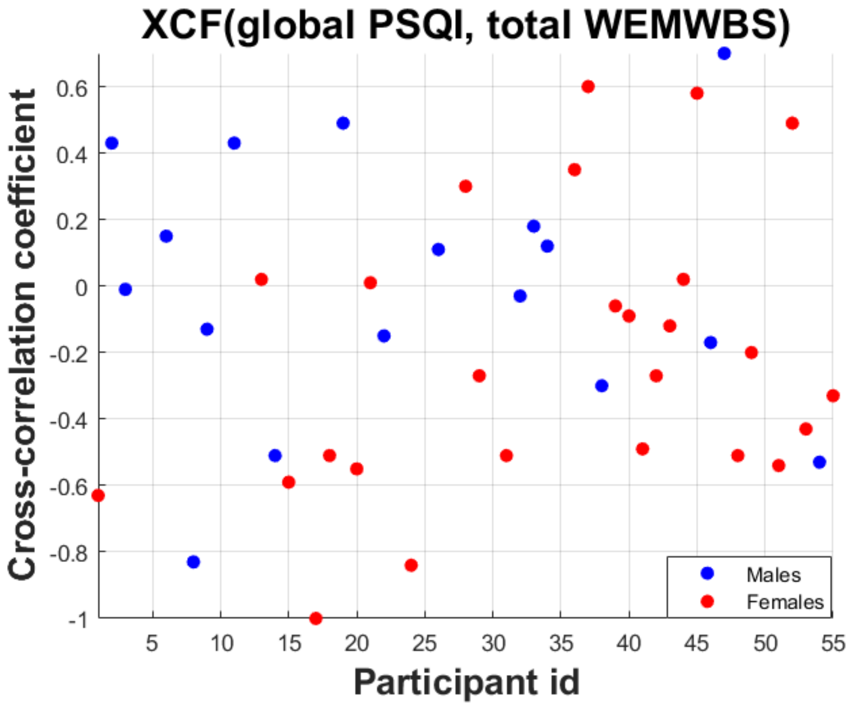

Figure S6: *Scatter plot with the cross-correlation coefficients (zero lag) between global PSQI and total WEMWBS across participants. XCF stands for cross-correlation coefficient.*

Exploring the relationship of actigraphy and PROMs further

Figure S7 builds on the same concept of gender-stratification presented in the previous section, this time using the actigraphy data to enable comparisons between males and females and explore how well these match self-reports in Figure S2. Specifically, we reported average sleep duration, wake-after-sleep-onset (WASO) and awakenings, and daily moderate-to-vigorous activity (MVPA) that have been extracted by processing the actigraphy data. To avoid any individual dominating the effect on these plots, we have first computed the averages per person before computing the underlying distributions presented with the violin plots. We remark that the sleep duration computed using actigraphy was indeed higher for women in general (matching self-reports in Figure S2 (a), (see Duration item).

There was greater variability in the WASO and awakenings (which collectively can be considered as sleep disturbances) for women, although overall men had somewhat more sleep disturbances on average. What's more, for males we observed there was a consistent pattern that all male participants, on average, experienced some sort of sleep disturbances and spent time awake in bed following sleep onset. Men engaged in more MVPA in general, with some women having very sedentary lifestyles as indicated by the actigraphy data. The findings for actigraphy-derived sleep efficiency in Figure S7 (d) contradict somewhat the self-reports presented in Figure S2 (a) (see Efficiency item) where women self-reported considerably worse sleep efficiency compared to men.

Figure S8 illustrates joint actigraphy-PROMs trajectories to visually inspect actigraphy-extracted information and PROMs over the course of the study for an indicative participant to get a feel for an individual's actigraphy-PROMs associations. We remark that for this participant (used as an example) we can see that their sleep duration was quite variable in the second and third times they wore the Geneactiv device and in general they were less physically active compared to the first time actigraphy data was collected. Therefore, intuitively this might explain the rise of PSQI and drop in total WEMWBS that was observed in September/October. This is only an example for a single individual, however this might serve well for the type of insights we might try to obtain with longitudinal actigraphy.

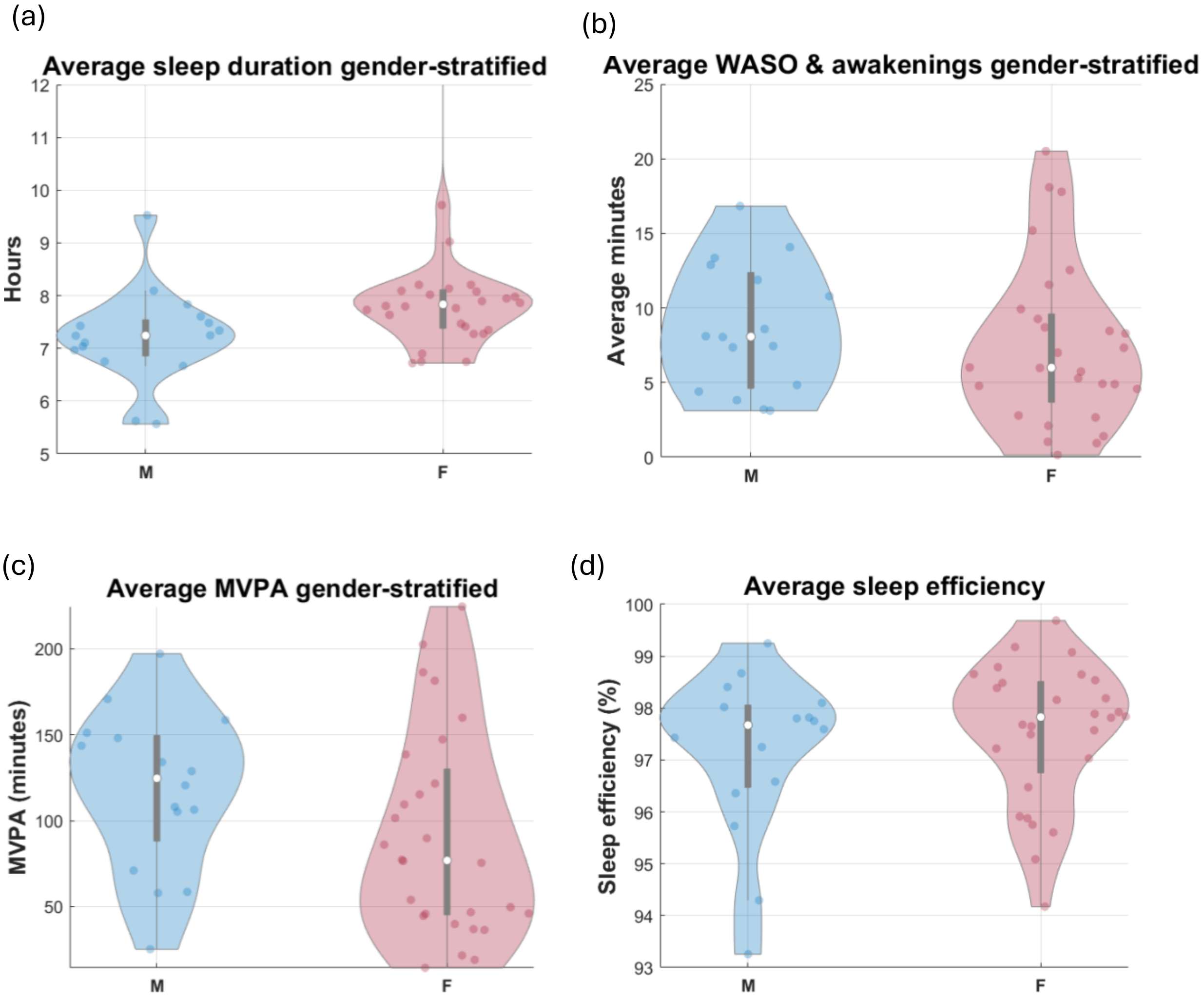

Figure S7: *Violin plots exploring gender differences in terms of actigraphy-extracted (a) sleep duration, (b) wake-after-sleep-onset (WASO) and awakenings, and (c) moderate-to-vigorous activity (MVPA). We pooled data from the different work sectors together before stratifying into males (M) and females (F), as denoted in the plots.*

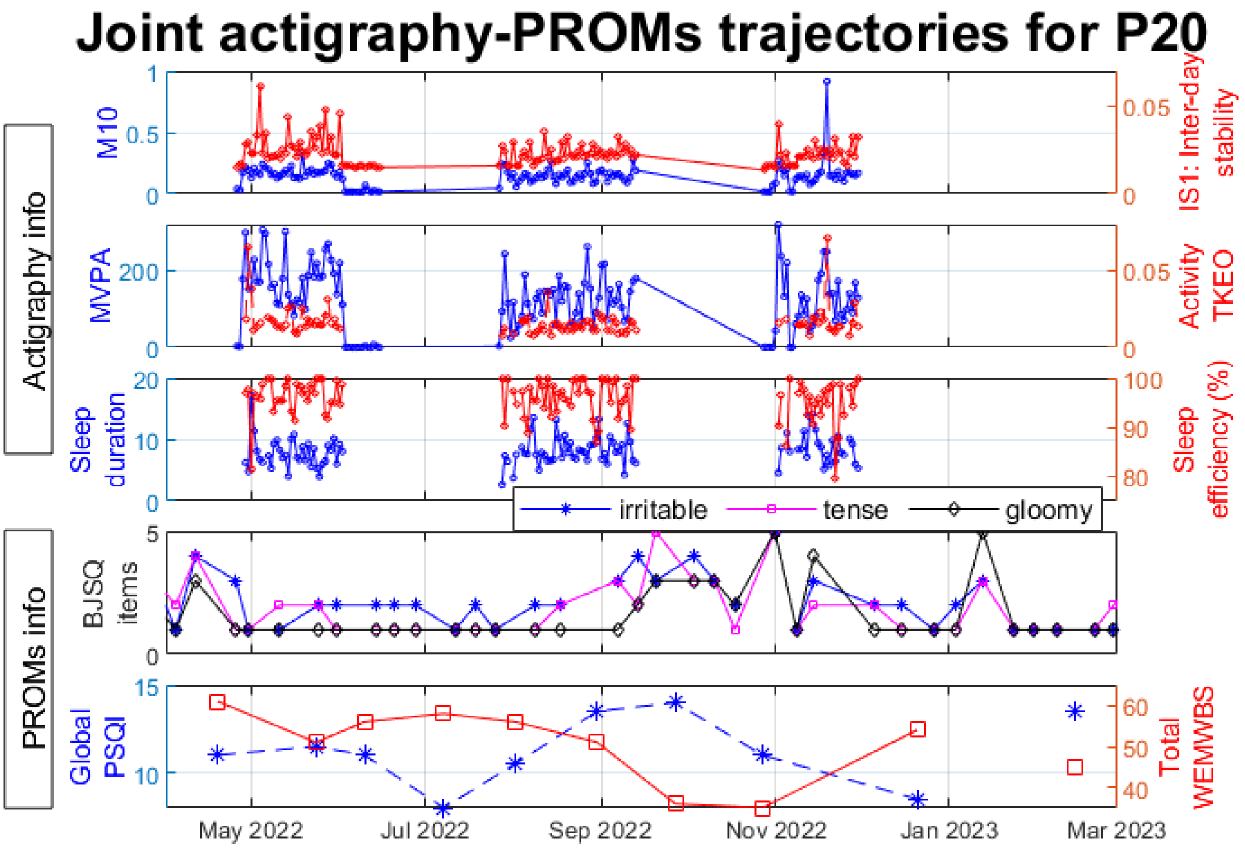

Figure S8. *Selected actigraphy extracted information (first three subplots) and PROMs (last two subplots) to illustrate trajectories for an indicative participant. The BJSQ items (fourth plot) have been collected weekly; the global PSQI and total WEMWBS monthly. Where presenting two axes in the same plot, the blue color corresponds to the left hand-side, and the red color to the right hand-side (hence the colors of the axes labels).*

Figure S9 serves to summarize the XCF between an indicative actigraphy measure (sleep efficiency) and overall questionnaire outputs. For convenience we have color-marked males and females, similarly to Figure S5. We note some strong associations for certain individuals, however neither the magnitude nor the direction of those relationships are universally consistent for either males or females. In a sense, this echoes the finding in Figure S5 where the self-reported scores on sleep (via PSQI) did not necessarily consistently translate in the self-reported scores on wellbeing (via WEMWBS). Importantly, we note there are strong associations of sleep efficiency and wellbeing for many participants (although the direction of the relationship is not consistent).

(a)

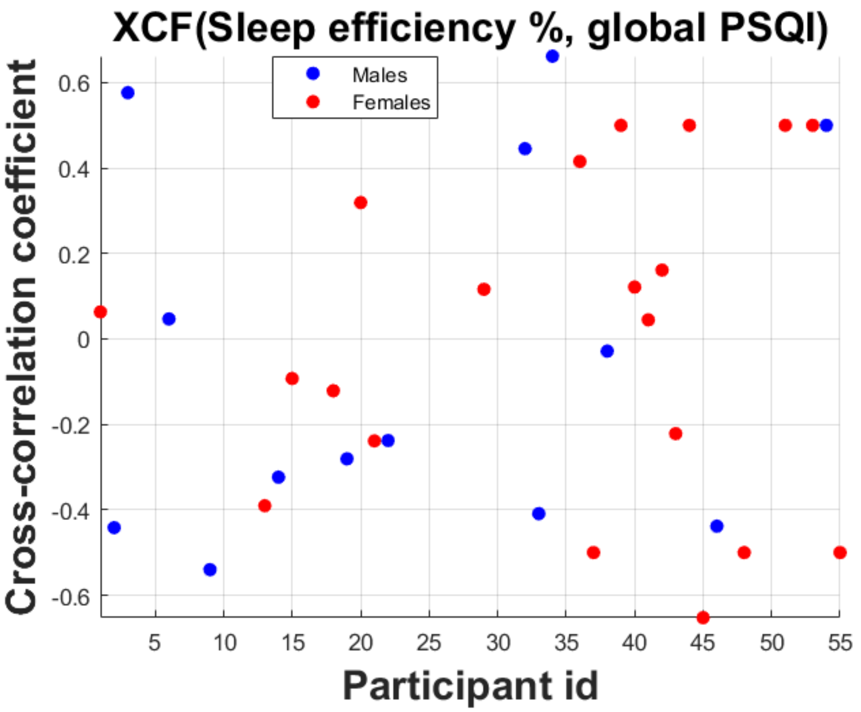

(b)

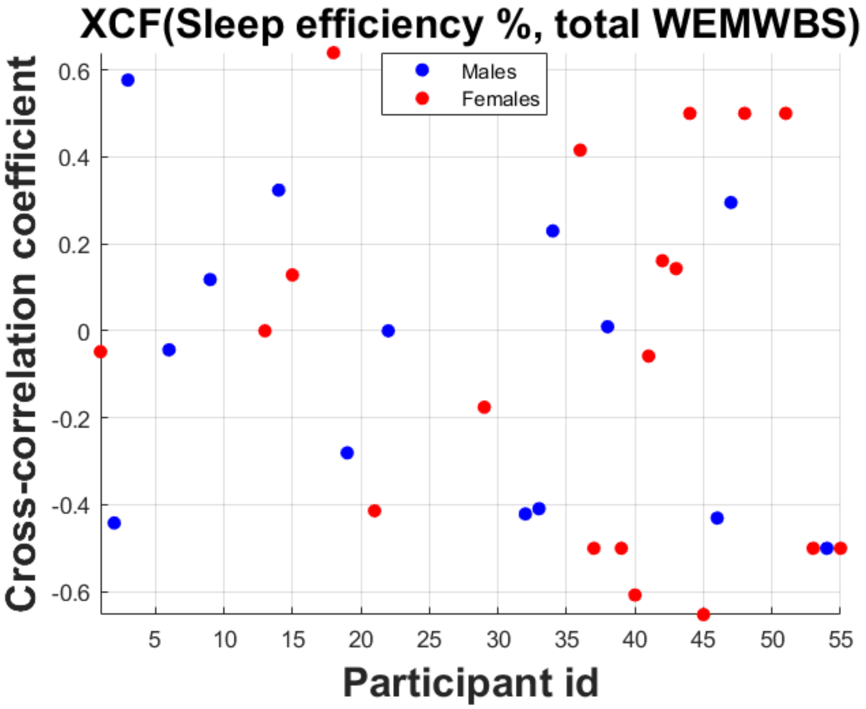

Figure S9. *Scatter plot with the cross-correlation coefficients (zero lag) of sleep efficiency with (a) global PSQI and (b) total WEMWBS, across all participants. XCF stands for cross-correlation coefficient.*

Further considerations and discussion

We have used short terms of the PROMs items in the figures presented herein (Figure S2) and the main manuscript for convenience in presentation. However, we emphasize that it is extremely important how the questions are phrased and the context within they are elicited: all findings should be carefully considered with the exact wording used in the original questionnaires. In that regard, we provided links to the exact wording that was used in the manuscript, along with a summary in the Supplementary Material titled "Questionnaires_in_SHAW.xlsx" file.

Longitudinal regular adherence and retention in studies is often challenging in practice, with more than half of the participants often discontinuing participation after a week [4]. In SHAW, adherence in completing PROMs was very good overall, and importantly remained fairly steady throughout the study: the running adherence for most participants was *consistently* between 50-80% (Figure S1). This is particularly promising given that there were no strong retention incentives for the study participants, as we elaborate on the main manuscript.

We explored whether there were differences when stratifying the data by gender. We found that, overall, there were mostly minor differences between males and females in terms of the baseline questionnaires, with a few exceptions potentially pointing to some nuanced perceptions (Figure S2). For example, women self-reported having less problems with sleep duration than men, a finding that was supported by the actigraphy data (Figure S5a). Women also self-reported that their sleep efficiency was considerably affected and much worse compared to what men self-reported, however actigraphy data suggest that on average the sleep disturbances and sleep efficiency derived was, if anything, somewhat better than men.

We found that when comparing the time series (e.g. pairwise comparisons across questionnaires), relationships were often less consistent than when using the baseline scores and computing the standard correlation coefficients. This highlights the underlying complexity of monitoring trajectories between questionnaire items longitudinally: for some participants we observed high positive XCFs and for some participants high negative XCFs for the same comparisons, as indicatively seen in Figure S5 exploring the association between global PSQI and total WEMWBS. Implicitly, this suggests the need to be exploring in more detailed personalized models to understand individuals' needs, and is in agreement with the research literature emphasizing that any potential interventions have to be tailored to match the specific circumstances of each individual [5,6].

The wrist-worn wearable devices were posted to participants using the standard UK mail service, where we had also included pre-paid envelopes for their safe return (for most participants this was three times, see Figure 1 in the main manuscript). Despite some initial reservations when designing the study about potential damages or losses of the devices through post, we had no problems: we found this worked very efficiently and cost-effectively to ship devices across the country. The only slight downside was that between the time of shipping the device and the time that participants actually put them on we had missing data (see Figure 1 in the main manuscript where always the first few days we had missing data).

References

1. Diggle, P. J., Heagerty, P. J., Liang, K.-Y. & Zeger, S. L. *Analysis of Longitudinal Data*. (Oxford University Press, Oxford, 2002).

2. Tsanas, A. *et al.* Daily longitudinal self-monitoring of mood variability in bipolar disorder and borderline personality disorder. *J. Affect. Disord.* **205**, 225–233 (2016).

3. Tsanas, A. *et al.* Clinical insight into latent variables of psychiatric questionnaires for mood symptom self-assessment. *JMIR Ment. Heal.* **4**, e15 (2017).

4. Pratap, A. *et al.* Indicators of retention in remote digital health studies: a cross-study evaluation of 100,000 participants. *npj Digit. Med.* **3**, 21 (2019).

5. McPhee, J. S. *et al.* Physical activity in older age: perspectives for healthy ageing and frailty. *Biogerontology* **17**, 567–580 (2016).

6. Michie, S., van Stralen, M. M. & West, R. The behaviour change wheel: a new method for characterising and designing behaviour change interventions. *Implement. Sci.* **6**, 42 (2011).
